# MSN, MWCNT and ZnO nanoparticle-induced CHO-K1 cell polarisation is linked to cytoskeleton ablation

**DOI:** 10.1186/s12951-021-00779-7

**Published:** 2021-02-12

**Authors:** Karmveer Yadav, Syed Azmal Ali, Ashok Kumar Mohanty, Eshwarmoorthy Muthusamy, Kesavan Subaharan, Gautam Kaul

**Affiliations:** 1 N.T. Lab-1, Division of Animal Biochemistry, ICAR-National Dairy Research Institute, Karnal, 132001 India; 2 Cell Biology and Proteomics Lab, Animal Biotechnology Centre, National Dairy Research Institute, Karnal, 132001 Haryana India; 3 Chemistry and Physics of Materials Unit, Jawaharlal Nehru Centre for Advanced Scientific Research, Bangalore, 560064 India; 4 Division of Germplasm, Conservation and Utilisation, National Bureau of Agricultural Insect Resources, Bangalore, 560024 India

**Keywords:** Apoptosis, Cell morphology, Cytoskeleton dynamics, ER-mediated phagocytosis and nanoparticles

## Abstract

**Background:**

The cellular response to nanoparticles (NPs) for the mechanical clue and biochemical changes are unexplored. Here, we provide the comprehensive analysis of the Chinese Hamster Ovary (CHO-K1) cell line to study cell behaviour following the exposure of mesoporous silica nanoparticle (MSN), multiwall carbon nanotubes (MWCNTs), and zinc oxide (ZnO) NPs.

**Results:**

Through the high-throughput proteomic study, we observed that the effect of NPs is alone not restricted to cell viability but also on cell polarisation. In the case of MSN, no drastic changes were observed in cellular morphology, but it upregulated chaperons that might prevent protein aggregation. However, MWCNT showed elongated cell appearance with numerous cytoplasmic vacuoles, and induce lamellipodia formation through actin polymerisation. The cytoskeleton remodelling was accompanied by the increased expression of Dlc-1, cofilin and Rac1 proteins. While ZnO NPs resulted in the rounded cell morphology along with nuclear abnormalities. The proteome analysis revealed that UBXN11 control cell roundness and DOCK3 leads to actin stress fibre formation and finally, loss of cell adhesion. It enhances the expression of catastrophic DNA damage and apoptotic proteins, which was unrecoverable even after 72 h, as confirmed by the colony formation assay. All three NPs trigger over-expression of the endocytic pathway, ubiquitination, and proteasomal complex proteins. The data indicate that ZnO and MSN entered into the cells through clathrin-mediated pathways; whereas, MWCNT invades through ER-mediated phagocytosis.

**Conclusions:**

Based on the incubation and concentration of NPs, our work provides evidence for the activation of Rac-Rho signalling pathway to alter cytoskeleton dynamics. Our results assist as a sensitive early molecular readout for nanosafety assessment. 
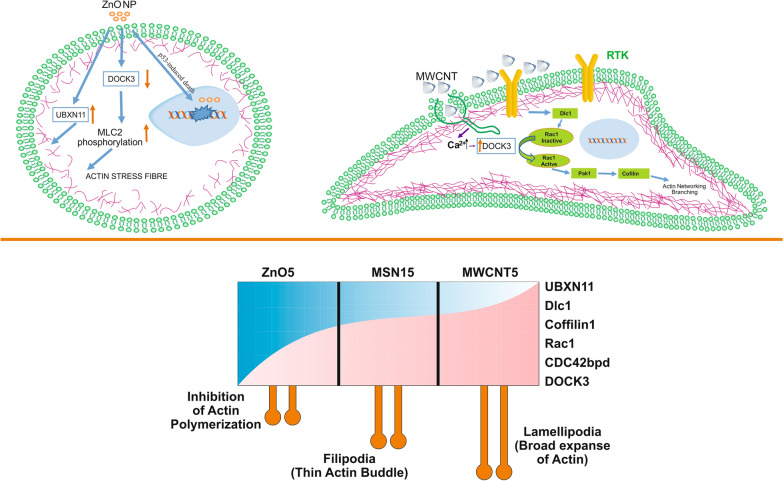

## Introduction

Nanotechnology has emerged as a multidimensional discipline getting utilised extensively in everyday life and biomedical field. It raises an urgent need to understand the safety of new-age materials in biological systems (liver, kidney, and lungs). High binding affinities of nanoparticles (NPs) leads to their accumulation in the target cell or organ [1]. It is not apparent how various NPs accumulation influences cell morphology, organelle shape, protein localisation, and interactions. However, their uptake, accumulation, and degradation are indulged with the plasma membrane, endoplasmic reticulum (ER), mitochondria, and lysosomes are incredibly dynamic. It alters their natural properties, e.g., structure composition and molecular instructions to acclimatise towards micro-environmental changes [2]. Uptake of NPs mainly occurs through the clathrin-mediated endocytosis pathway, and clathrin vesicles (CVs) fused with the endocytic compartment. The fusion of CVs can regulate cell migration via the extracellular matrix, which involves cytoskeletal components at the protrusive edge and signalling molecules [1]. This correlation sheds light on the cytoskeleton connection point and its contribution to the cell internalisation process. Altogether, it is crucial to understand cytoskeleton proteins role in regulating cellular adaptations, proliferation and morphology, that are poorly studied to NPs exposure.

NPs use in cancer treatment is rapidly growing, due to the multidimensional mode of action that can be activated by the system, which determined various factors, including the type of NPs and their properties [3, 4]. Earlier, the mechanisms of action associated with iron NPs anti-tumour activity are involved in apoptosis and anti-angiogenesis [5, 6]. Mesoporous silica nanoparticle (MSN) and zinc oxide (ZnO) NPs have got limelight attention in the well-established nano-biomedical application and the nascent nano-agricultural domain, due to their unique properties and low production cost [7–9]. MSN has a large pore volume, uniform porosity, and preferentially excellent biocompatibility, making them an essential compound for drug delivery system [10]. Additionally, silica and zinc provisionally approved as "Generally Recognised as Safe" (GRAS) materials by FDA and allowed to be used as a food additive and in cosmetics (US FDA GRAS report) [11].

Significant biomechanical effects can be trigged by carbon nanotubes, particularly multi-wall carbon nanotubes (MWCNTs), due to their resemblances to microtubules that induce their interaction in the cells [12, 13]. Since the growing number of anticancer drugs targets the microtubules and actin filaments, getting to the bottom of the relationship between MWCNTs and cytoskeleton could be the significant step for putting bio nanotechnology closer to the next generation of biocompatible particles for chemotherapy [13, 14]. Other NPs such as TiO2, SiO2, and hydroxyapatite nanoparticles have been shown to induce microtubule network damage and inhibit cell migration [15].

In the current study, we used a high-throughput proteomic approach to revisit past research, such as NPs uptake and cell proliferation, and suggest a new cellular organisation model and the link between ER and NPs uptake. Our findings reveal context depended morphological alteration, decrease in cell viability and prevention of cell adhesion. However, cell viability assays were not sufficient to project the reported alterations so that there is an urgent necessity for a more decisive way to assess the biological impact of NPs treatment. The morphological assessment showed that the adverse effect of ZnO NPs makes the cell rounded and unable to adhere. MWCNT can induce cell enlargement and more effective spreading. While, in the case of MSN, cells revealed a comprehensive ability to attach to the surface. The present findings proved the direct correlation between the cytoskeleton and altered cell morphology.

## Materials and methods

### Nanoparticles suspension preparation and characterisation

Zinc oxide suspension (Cat. 721077-100G) and Multiwalled carbon nanotube (Cat. 659258-2G) were purchased from Sigma Aldrich. MSN synthesis has been previously described by Rawat N. et al*.* 2016 and Bara N. and Kaul G., 2018. First of all, nanoparticles were diluted with HAM's F-12 culture medium (Genetix Biotech Asia Pvt. Ltd., India) followed by sonication by using the ten cycle (pulse ON 15 s and OFF 15 s) in cooling condition to avoid aggregation. The particle dispersion and the average hydrodynamic size were measured by Malvern Zetasizer (NanoZS90, Malvern Instruments Ltd., Malvern, UK). Transmission electron microscopy (TEM) is among the foremost methods to analyse nanoparticles structure and dimension. The shape of ZnO, MWCNT, and MSN were observed under TEM (Tecni, Amsterdam, Netherlands) at a minimum of four fields of view and 200 kV an accelerating voltage.

### CHO-K1 cell culture and nanoparticles exposure protocol

The CHO-K1 cell line was obtained from National Centre for Cell Science (NCCS), Pune, India. The CHO-K1 cells were cultured in Ham's-F12 culture medium containing 10% fetal bovine serum (FBS) (HyClone, South Logan, UT) and penicillin (100 U/mL) streptomycin (100 μg/mL) at 37 °C with 5% CO_2_ humidified atmosphere (NuAire, USA). ZnO, MWCNT, and MSN were dissolved in Ham's-F12 medium at 2 mg/mL concentration followed by sonication for 15 min. Dissolved NPs were diluted were with Ham's-F12 medium up to the indicated concentration. After dilutions, NPs were vibrantly vortexed for 30 s prior treatment to prevent NPs agglomeration.

### Cell uptake and TEM imaging of nanoparticles

The cellular uptake of nanoparticles was estimated, 1 × 10^5^ cells were seeded in a T75 flask, followed by overnight incubation at 37 °C with 5% CO_2_ to favour the cell attachment. On the next day, the cells were treated with ZnO1, ZnO5 (1 and 5 µg/mL, hereafter it will be used as ZnO1 and ZnO5), MWCNT5 (5 μg/mL, hereafter it will be used as MWCNT5) and MSN15 (15 μg/mL, hereafter it will be used as MSN15) for 24 h. The cells were washed carefully thrice with PBS and prefixed for 1 h in the fixative medium (2.5% glutaraldehyde and 2% paraformaldehyde in 0.1 M PBS (pH 7.4)), then cells were removed by scraping and washed twice with PBS. The cell ultra-sectioning was performed after fixation by using, 1% osmium tetroxide for one hour. Dehydration was done with various ethanol concentration (30, 50, 70, 80, and 95%) for 10 min at each gradient, and end up with three dehydration cycle using 100% ethanol for 10 min each. Furthermore, samples were embedded in Spurr resin and kept at 68 °C for 15 h to get polymerised after that ultrathin sectioning was performed by ultra-microtome and visualised under TEM without staining.

### Cell cytotoxicity assay

#### MTT assay

We used MTT to check the cell proliferation of NPs treated CHO-K1 cells. 2 × 10^5 ^cells/100 μl/well were seeded in 96-well plates and kept for 24 h incubation. The medium containing different dilution of each NPs (0 [control] or 1, 2, 5, 10, 20, 50, or 100 µg/mL) was prepared and added to the cells in triplicate. After 24 h of MSN, MWCNT, and ZnO NPs exposure, 15 μl of freshly prepared MTT [5 mg/mL of Thiazolyl Blue Tetrazolium Bromide (MTT Powder, Sigma-Aldrich Cat. # M2128)] in Dulbecco phosphate buffer saline (DPBS) was added and further incubated for 4 h at 37 °C. The cells were gently washed with PBS, and resultant formazan crystals were dissolved in 100 μL of DMSO. After shaking for 1 min at 240 rpm, absorbance was finally read at 570 nm on an Infinite M200 multi-well plate reader (Tecan, Durham, USA). The cell viability percentage of the treated cells was calculated relative to the untreated control cells, which was supposed to be 100%.

#### WST-8 assay

In the present study, WST-8 assay was picked because of two main reasons (i) to avoid the interference in MTT reduction assay cause by MWCNT and (ii) the reduced product of WST-8 assay is more soluble in culture media than MTT [16–18]. The WST-8 assay kit (Cayman Chemical, Cat. #10010199) was used to determine the different cytotoxic concentration of NPs, as per the manufacture protocol. CHO-K1 cells (2 × 10^5 ^cells/100 μL/well) were seeded in 96-well plates for 24 h and exposed with different concentration of MSN, MWCNT and ZnO NPs in triplicate. After 24 h, 10 µL of WST-8 solution were added to nanoparticle treated cells, and the plates were incubated at 37 °C for 2 h. The absorbance was measured at 450 nm using a Tecan multi-plate reader.

#### LDH Assay (Necrotic cell death analysis)

CHO-K1 cells were plated in culture plates (96-well) at 1.2 × 10^5^ cells/100 μL/well cell density and incubated for 24 h. After that, cells were treated with different concentrations of MSN, MWCNT, and ZnO NPs for 24 h. LDH release used the QuantiChromTM LDH cytotoxicity assay (C2LD-100, BioAssay System, CA, USA) following the manufacturer's protocols. Then, 160 μl of reagent solution was added and incubated for 10 min at 37 °C. Optical density was read at 500 nm by a microplate reader.

#### Trypan blue assay

Trypan blue, which is accumulates in dead cells but excluded from live, is one of the most regularly used procedures for cell viability assessment [19–21]. An identical set of plates were set up for the trypan blue assay, and cells were seeded and treated similarly to that defined in the above assay. After incubation for 24 h at 37 °C, 50 µL of sterile trypan blue solution (0.4%) was added a final concentration of 0.05% to each well and further incubated at 37 °C for 10 min. After three times washing with ice-cold PBS, 200 µL of the SDS solution 1% (w/v) was added, and finally, absorbance was read at 590 nm using a microplate reader.

#### Colony formation assay

100 cells/well were grown in 6-well plates. Initially, the plates were kept for three days in normal after that treated with MWCNT5, MSN15, ZnO1 and ZnO5 for 24 h. The medium was replaced at every 24 h time intervals. After three days of nanoparticle treatment, colony formation of CHO-K1 cells were detected. The cells were then prefixed with 4% paraformaldehyde, followed by staining with crystal violet (0.1% w/v). Colonies were calculated if they have a minimum of 15 cells. Images were captured with a phase-contrast microscope equipped with a digital camera.

### Effect of NPs on cell morphology and symmetry (Microscopically)

**Fig. 1:**
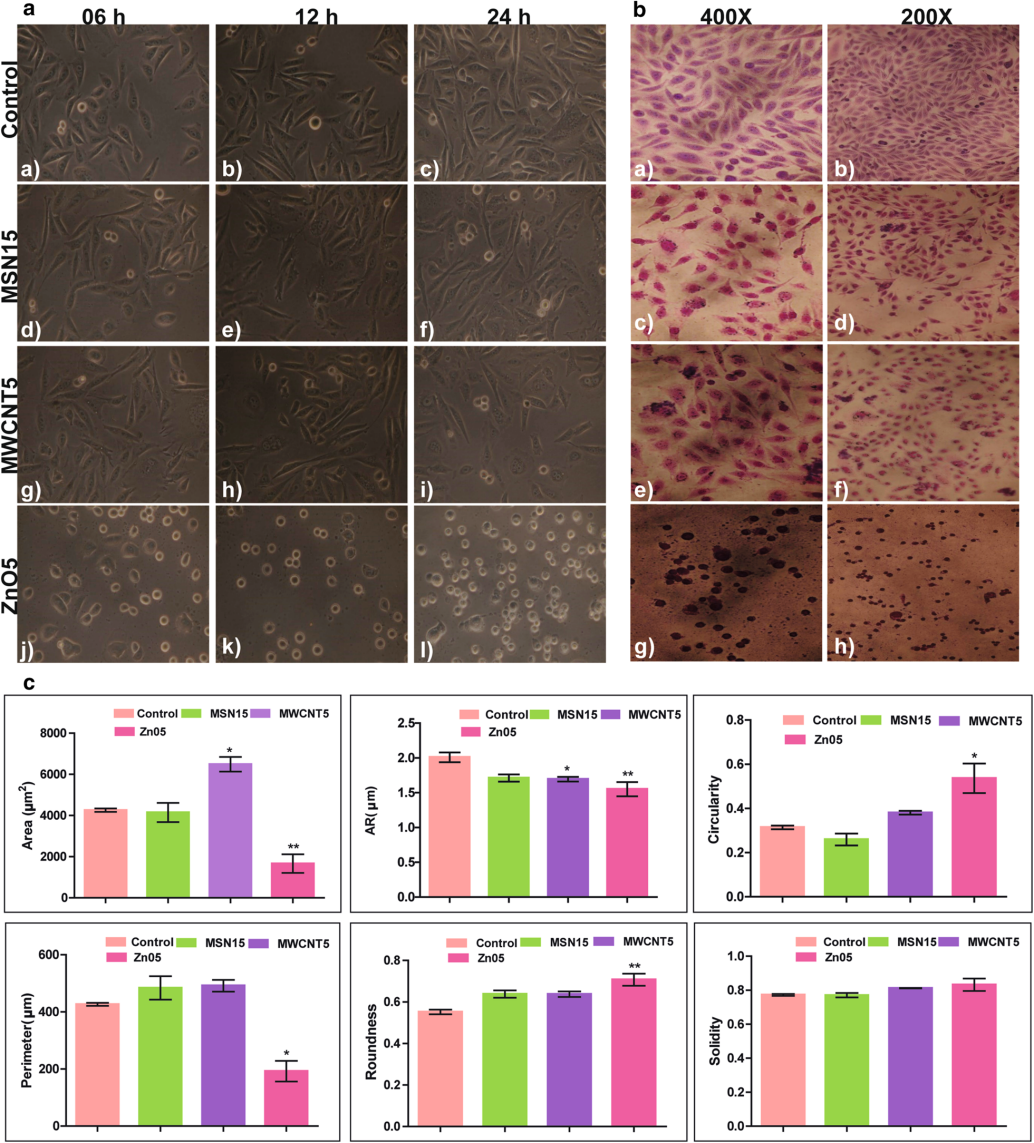
**A** Effect of NPs on CHO-K1 cells morphology and symmetry under a bright-field microscope: CHO-K1 cells were seeded in 6-well plates and NPs treatment after 24 h stabilisation of cells. CHO-K1 cells treated with ZnO5, MWCNT5, MSN15 and control cells. Photographs were taken after 6 h (**a**, **d**, **g**, and **j**), 12 h (**b**, **e**, **h**, and **k**) and 24 h (**c**, **f**, **i**, and **l**) of treatment without stain. **B** CHO-K1 cells treated with MSN15 (**c** and **d**) MWCNT5 (**e** and **f**) ZnO5 NPs (**g** and **h**) and control cells (**a** and **b**). After 24 h treatment cells were stain with wright stain. 200X and 400X magnification. **C** Morphological changes of CHO-K1 cells analysed at 24 h post-treatment of MSN15, MWCNT5, and ZnO5 NPs: (i) Area, (ii) Aspect Ratio (iii), Circularity, (iv) Perimeter, (v) Roundness, and (vi) Solidity measurements were taken. The results indicate that CHO-K1 cells on the MWCNT5 treatment surface area increase significantly. The ZnO5 treatment decreases the surface area. The aspect ratio of CHO-K1 cells initially began close to 2, but over 24 h treatment, the ZnO5 decreased significantly higher than the MSN15 and MWCNT5. ZnO5 show highest circularity, whereas MSN15 show lowest circularity compared to control. MWCNT5 treated cells show more elongated or cuboidal morphologies indicated by lower aspect ratios and similar circularities, whereas the ZnO5 treated surface moved toward a circular morphology

CHO-K1 cells were plated in Ham's F-12 medium at low density (1 × 10^3^ cells/well in 6-well plates). When single cells had stretched into well-defined colonies. After 24 h, the medium containing MSN15, MWCNT5, and ZnO5 NPs were added and monitored individual cell clusters over 24 h. The images were taken using an Olympus microscope (40 × objective) at time intervals showed in Fig. 1a. Then, the cells were fixed with 95% ethanol and stained by 0.4% wright stain. Image processing was done using the ImageJ image software ((Wayne Rasband, USA)) to obtain cell shape parameter (cell symmetry) within an individual image and calculated their area, circularity, roundness, solidity, aspect ratio, and perimeter. A circularity index was measured as 4π(area/perimeter^2^) (= 1 for a circle) for the individual cluster.

### Nanoparticles treatment and proteomic sample preparation

For proteomics sample preparation, the total cell count of 2 × 10^5^ CHO-K1 cells was seeded in T75 flasks and incubated for 24 h to achieve 70% confluence, followed with the addition of 15 mL fresh culture media containing separately, NPs of MSN15, MWCNT5 and ZnO5 for 24 h. Before the addition, all the NPs were sonicated by keeping in ice for 15 min. After the treatment, the media was removed, and cells were rinsed three times with DPBS. The obtained cells were resuspended in 1 mL of TEAB lysis buffer (50 mM Triethylammonium bicarbonate, 1% sodium dodecyl sulfate, and 1 mM PMSF, pH 7.4) containing fresh protease and phosphatase Inhibitor tablet (Cat no. A32959, Thermo Scientific). The cells were given a cold shock in liquid nitrogen followed by ten cycles of sonication with 3 min interval between each cycle (pulse ON 7 s and OFF 15 s). Sonicated cells were centrifuged for 15 min at 2500 g, and the resultant supernatant has been transferred to the new microtube. The bicinchoninic acid (BCA) assay was used to determine each sample's protein concentration, and to check the quality of protein, equal amounts of individual samples (30 µg) were separated on SDS-PAGE. The equal amount of protein samples was aliquoted, lyophilised, and stored in − 80 °C till further use.

### Protein digestion, labelling, and RP-HPLC fractionation

Each group lyophilised protein sample (140 μg) was reconstituted in 20 μL containing 1 M triethylammonium bicarbonate (TEAB) with 2% sodium dodecyl sulphate (SDS). The samples then reduced to 50 mM tris-(2-carboxyethyl) phosphine (TCEP) for one-hour at 60 °C. The cysteine residues were blocked in the dark at room temperature using 200 mM iodoacetamide (IAA) for 30 min. The proteins were initially mildly incubated for digestion using trypsin/Lys-C (Mass Spec Grade, Promega, Madison, WI) at 1:50 (trypsin/Lys-C: protein ratio) for 3 h at 37 °C followed with fully digestion by trypsin (Gold Mass, Promega, Madison, WI) 1:30 (enzymes: protein ratio) at 37 °C for 16 h. Individual group digested peptide samples were labelled with iTRAQ channels following the manufacture protocol (AB Sciex), and the reaction was stopped with the addition of deionised water. The labelling sequence for samples are as follows: control 114; MSN: 115; ZnO: 116; MWCNT: 117. The pooled labelled sample was fractionated by RP-HPLC (Agilent 1100) using analytical column (Grace Smart RP, C18 (150X4.6 mm), 5 μm). The mobile phase A (10 mM TEAB) and mobile phase B (10 mM TEAB in 90% ACN) was used respectively for the peptides separation using following linear gradient: 0 to 2% in 5 min, 2 to 60% in 60 min, 60 to 100% in 25 min and then 100 to 2% in 2 min. The samples were divided into ninety-six fractions and were recollected back using concatenated pooling into eight fractions. The final collected eight fractions were lyophilised and again reconstituted in 0.1% formic acid (FA). The samples were used for desalting utilising commercially available C18 stage tip column (Ziptip, Millipore, USA). Eluted peptides were lyophilised and re-dissolved in 0.4% FA and used for MS/MS spectra generation.

### LC–MS/MS analysis

The iTRAQ labelled samples were subjected to Q-TOF mass spectrometer (Bruker Daltonics, Germany) equipped with nano sprayed captive source (using 1850 V, Bruker Captive tip), the acquisition parameters were used as per previously described protocol [22]. Briefly, to increase the ions intensity and sensitivity, the captive tip and glass capillary was sprayed with 100% ACN using a nano booster. The peptides were injected using the trap column (Bruker Magic C18 AQ, 0.1 × 20 mm, 3 μm, 200 Å) connected with nano-LC (Nano-Advance, Bruker, Germany) containing C18 reversed-phase resin nanoanalytical column (Bruker Magic C18 AQ, 0.1 × 150 mm, 3 μm, 200 Å) at 400 nL/min flow rate. The continuous gradient of 5–45% ACN over 90 min with a total time of 135 min in 0.1% FA as Solvent A and 100% ACN in 0.1% FA as Solvent B. The MS instrument was operated in Data Dependent Acquisition (DDA) mode to automatically identify the MS and MS/MS spectra, precursors and related transitions. For the MS spectra scan, the precursor ion window was 300–1800 (m/z) with the resolution of 75 K at 600 m/z. The five most abundant parent ions were fragmented in collision-induced dissociation (CID). The duty cycle fixed time was 0.5 s together with 1 min exclusion filter for abundant ions. The singly charged and the unknown state ions were excluded from being selected for MS/MS (otof processing software, Bruker Daltonics). Each sample analysis was done in duplicates.

### Raw files data analysis

The created raw (.d) libraries were converted to mzML format using MSconvertGUI. The mzML data were examined using the Trans-Proteomic Pipeline (TPP, ver. 5.1.0) with added combined in-house UniProt databases, *Cricetulus griseus* (Hamster), *Mus musculus* (Mouse) and *Rattus norvegicus* (Rat). The complete database was also augmented with common sequences of contaminant with identical reversed decoy proteins sequences. We utilised multiple search engines, as describes previously [23]. Multiple search engines were used for the peak identification and peptide assignment using Tandem, SpectraST, and Comet. We kept all parameters similar to all the search engine that includes enzyme Lys-C and trypsin, and two miss cleavage permitted, N-terminus and Lys for iTRAQ reporter ions, Cys for carbamidomethyl as fixed modifications. While oxidation of Met, Gln-pyro Glu and Glu-pyro Glu as variable modification. Seven amino acid minimum lengths were kept for peptide identification. Mapped were scored using the PeptideProphet and ProteinProphet algorithms to calculate the probabilities score. The precise PeptideProphet model was used for high confidence peptide identifications to improve the probability of peptides. Additional protein confirmation step was performed using together Peptide Prophet and Protein Prophet scores, through filtering the proteins containing more than two peptides with 95% or higher probability. All the search engine results were combined, and last validation statistics were implemented using the iProphet. It uses the PeptideProphet output information and calculates the new, more stringent score for unique peptide sequence [24]. In this way, we combined the results from three search engines using the iProphet scoring algorithm and selected all the proteins with more than 0.95 cutoffs.

### Bioinformatics analysis and network construction

The examination and forecast of proteins interactions networks were prepared using differentially expressed proteins (DEPs). The high confidence interaction (overall 0.90 scores) among the proteins were collected from STRING [25] and annotated them in the Cytoscape platform together in combination with the Reactome database [26]. The determination of NPs induced biologically relevant pathways, the KEGG database [27] (Kyoto Encyclopedia of Genes and Genomes) search was performed using the whole protein sequence, which was retrieved using the in-house prepared python script. Further, the GOEAST [28] (Gene Ontology Enrichment Analysis Software Toolkit) was used to calculate the over/under enrichment of Gene Ontology (GO) category. The program used the binomial based probability scoring statistics for the calculation of *p*-values and e-values. We set the threshold of 0.05 cutoff for selection of the differential fold enriched GO term.

Next, NPs-dependent enriched proteome specific parent-terms analysis was performed using the ClueGO and BiNGO for the functionally connected linkage. Using the kappa score based statistics model provides the degree of relationship among the pathways associated with significant relevant overlapping proteins and GO terms. The threshold 0.4 Kappa statistics was set, and above that, all the terms connections were selected to illustrate the significant interactome. The analysis uses the following parameters minimum three and maximum eight-level for GO tree, minimum 3% of genes of GO term/pathway, Benjamini and Hochberg test with the two-sided hypergeometric distribution of corrected FDR level < 0.05 significance.

### In vitro wound-healing assay

CHO-K1 cells were plated in 6-well plates and kept in an incubator for 48 h. The monolayer was scraped using a p200 pipet tip to make a "scratch" and washed with DPBS to remove cells debris. CHO-K1 cells treated with MSN15, MWCNT5, ZnO1, ZnO2 and ZnO5 NPs. The images were captured at hour 0, 6, and 24 h, respectively, after the wound was created. Images were analysed by ImageJ software. The area of the uncovered surface was calculated immediately after wounding and for the experiment's entire duration. The relative wound closure potential was calculated the ratio between the surface area of the wound for various time points and the initial wound's surface.

### Statistical analysis

All data were analysed using GraphPad Prism 5.01 (GraphPad Software, San Diego California USA, http://www.graphpad.com). The significance of the difference between mean values calculated by one-way analysis of variance (ANOVA) with Tukey's posthoc test. The data were represented as mean ± SD, and the value of p < 0.05 was considered statistically significant.

## Results and discussion

### Structural characterisation of NPs through TEM and DLS

**Fig. 2:**
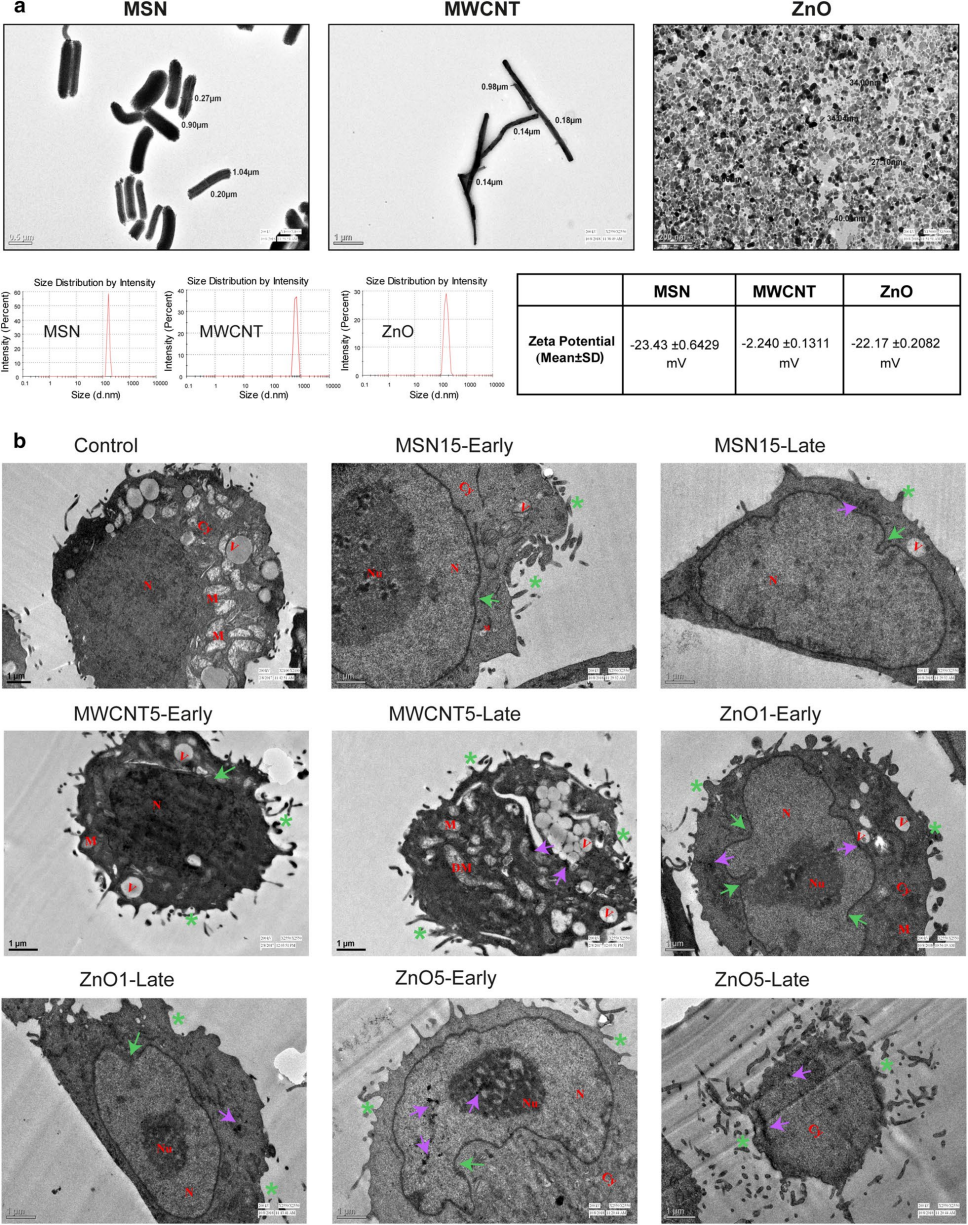
**a** Characterisation of the MSN, MWCNT and ZnO NPs size. Transmission electron microscope (TEM) images of ZnO (diameter: ~ 44 nm) with a spherical shape, MSN with rod-like structure (width: ~ 200 nm, length: ~ 1040 nm), and MWCNT with rod-like structure (width: ~ 140 nm, length: ~ 980 nm). Below Figure represents the dynamic light scattering (DLS) profiles of 198 nm ZnO NPs, 667 nm MSN and 1474 nm MWCNT. Zeta-potential values were measured using DLS represented in the table with mean + SD. **b** Cellular uptake of MSN, MWCNT and ZnO NPs: CHO-K1 cells exposed with MSN15, MWCNT5, ZnO1 and ZnO5 NPs concentration induced both apoptotic and necrotic cell death. Apoptotic cell with typical nuclear membrane dilatation (green arrowheads) and blebbing (asterisk). Moreover, we observed MSN in the cytoplasm in the late-stage MSN treated cells. The necroptotic cell death was preceded by intensive cytoplasm vacuolisation (v), disrupted cellular membrane, and cytoplasmic swelling (MWCNT early and late-stage). MWCNT late-stage treated cells, also showing crescent-like vacuoles and damaged mitochondria. ZnO1 and ZnO5 lead apoptotic cells along with nuclear condensation (N), nuclear membrane dilatation (yellow arrowheads) and blebbing (asterisk). ZnO1 and ZnO5 NPs were observed in the nucleus, and no other particle found in the nucleus. In addition, irregular nucleus (invagination of the nuclear membrane) shape was observed, especially ZnO1 and ZnO5 treatment. Violet arrows indicate the presence of nanoparticles. Abbreviations: Cy, cytoplasm; N, nucleus; Nu, nucleolus; V, vacuoles; DM, damaged mitochondria

The TEM analysis of MSN and MWCNTs revealed long rod shape in structure, while ZnO NPs found a spherical structure (Fig. 2a). The identified size of MSN (width: ~ 200 nm, length: ~ 1040 nm), MWCNT (width: ~ 140 nm, length: ~ 980 nm), and ZnO NPs (diameter: ~ 44 nm) were shown in Fig. 2a. The TEM images implied that these NPs tend to cluster, signifying the agglomeration property. To identify the reason, we performed dynamic light scattering (DLS) analysis of NPs and found average hydrodynamic size of ZnO, MSN, and MWCNT NPs was ~ 198 nm (Fig. 2a with zeta potential -22.2 ± 0.13 mV), ~ 504 nm (Fig. 2a with zeta potential -23.8 ± 0.64 mV) and ~ 1561 nm (Fig. 2a with zeta potential -2.36 ± 0.20 mV) respectively, that is nearly three times bigger than the NPs observed size in TEM. This suggests that NPs could agglomerate in different medium during exposure to the biological system. It is also in correspondence to the previous study that demonstrates NPs agglomeration in different cell culture media [29]. Another study reported the different hydrodynamic diameter of NPs in a changing environment [30]. Therefore, our approach of using unaltered ZnO, MSN, and MWCNTs could better display the respective impact of NPs in a physiological environment, with the diverse dimension of populations (size and shape) entering to the CHO-K1 cells (Additional file 1: Figure S1).

Subsequently, to precisely demonstrate the pragmatic effects of NPs exposure on CHO-K1 cells, we need to verify if the ZnO, MSN, and MWCNTs could be internalised into the cells. TEM analysis showed (Fig. 2b) that NPs materialised within the plasma membrane of the cells. It seems that they are majorly localised in large vesicles in the cytoplasm. Therefore, we confirmed that NPs could penetrate the CHO-K1 cells to modify the intracellular biological milieu potentially [31, 32]. In the case of MSN15 (15 μg/mL, hereafter it will be used as MSN15) localised in the cytoplasm as free nanoparticles, single-membrane bounded vesicles as well as multivesicular vacuoles. MWCNT5 (5 μg/mL, hereafter it will be used as MWCNT5) treatment also showed the presence of crescent shape vacuoles and bi-layered membrane-bounded autophagic vacuoles with cell debris (Fig. 2b). Besides, more spread and filopodia were observed after exposure to MSN15 and MWCNT5. Quantitation of the increase in cell size is summarised in Fig. 1c. If early ultrastructural changes were evident and to ascertain if the increase in cell size resulted from vacuolisation or widespread proliferation of organelles. Although the cell components, such as mitochondria, Golgi, and endoplasmic reticulum, can be seen to have increased in number, they have not increased in size except mitochondria. The increase in cell size and the fact that the cell number did not increase in the treated cultures suggests that MWCNT5 inhibits the mechanisms controlling cell replication initiation.

No other particles except ZnO NPs were observed in the nucleus. Besides, irregular nucleus (invagination of the nuclear membrane) change in shape was observed, especially on ZnO1 and ZnO5 (1 and 5 μg/mL, hereafter it will be used as ZnO1 & ZnO5) NPs treatment. Our data suggested that ZnO NPs primarily affect cytostatic properties, followed by a chain of secondary interactions resulting in cell death. This result encouraged to elucidate the mechanism of apoptosis and catastrophic DNA damage due to ZnO NPs-induced cytotoxicity in CHO-K1 cells.

### ZnO, MWCNT, and MSN associated cytotoxic response in CHO-K1 cells

**Fig. 3:**
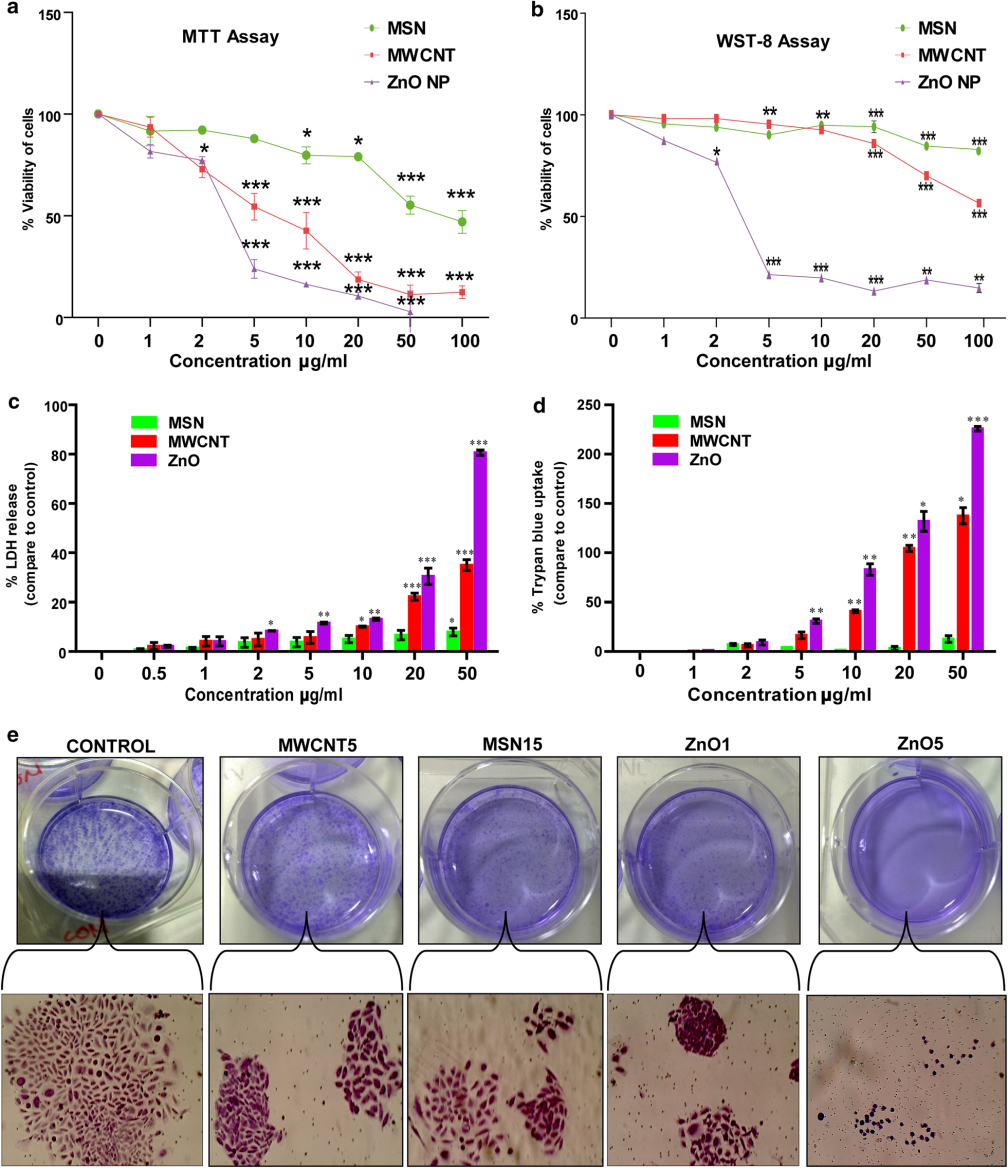
**a** Effect of MSN, MWCNT, and ZnO NPs on the CHO-K1 cell line: Cytotoxicity was measured by MTT (**a**), WST-8 (**b**), LDH release assay (**c**), and Trypan blue uptake (**d**) after treatment with ZnO, MSN, and MWCNT. CHO-K1 cells were cultured in Ham's-F12 culture medium for 24 h and then treated with different concentrations of NPs for 24 h. Cell viability was found to decrease with increasing concentration. The results were represented from three independent experiments as % of cell viability (mean ± SD) versus control cells (100%). Statistical analysis was calculated by one-way ANOVA with Tukey’s posthoc test (*p < 0.05, **p < 0.01, ***p < 0.001). **e** Nanoparticles induced colony formation inhibition of CHO-K1 cells: CHO-K1 cells were seeded in 6-well culture plates for 3 days and exposed with MWCNT5 (5 μg/mL), MSN15 (15 μg/mL), ZnO1 (1 μg/mL) and ZnO5 (5 μg/mL) for 24 h. The medium was replaced at every 24 h time intervals. After three days of nanoparticle treatment, colony formation of CHO-K1 cells were detected by crystal violet staining. Images were captured by a phase-contrast light microscope. 20X magnification

The MTT, cell doubling, WST and LDH-based assays were performed with a wide range of NPs concentration (1–100 µg/mL) during the 24 h period. This selected dose range compatible with the individual NPs to assess the toxicity in vitro [33, 34]. The cell proliferation assay indicated dose-dependent cytotoxicity, with less toxic effects of MSN than MWCNT and ZnO NPs. In the MTT assay, MSN, MWCNT and ZnO NPs showed 12%, 45%, 76% decreases in cell proliferation, respectively in comparison to the control (*P* < 0.01) at 5 µg/mL concentration (Fig. 3a). Simultaneously, the WST-8 assay indicated 10, 5, 78% diminished cell viability (*P* < 0.05) at 5 µg/mL concentration of MSN, MWCNT and ZnO NPs, respectively (Fig. 3b). Our findings were following the previous study reported by Wörle-Knirsch et al*.* showed that after 24 h treatment with carbon nanotubes (CNTs) to A549 cells, approximately 50% cell viability in the MTT test, whereas no cytotoxic effect was observed in the WST-1 assay [35]. Next, we assessed the effect of NPs induced necrosis in CHO-K1 cells using LDH assay because of the interfering effect (dose-dependent increase in absorbance) of MWCNT in the above assay. It demonstrated a significant increase in LDH level with the increasing concentration of MWCNT. At the concentration of 5 and 50 µg/mL, MWCNT increased the release of LDH around 6% and 35%, respectively, compared to the control. At the same concentration, ZnO NPs raise the LDH level around 12 and 80% respectively, whereas MSN 4% and 8% increase in the LDH level (Fig. 3c). It has been repeatedly acknowledged that NPs interact with reagents (MTT, WST-8, and LDH) to produce variable outcomes through dye-NPs binding or of the by dye-dye product adsorption [36, 37]. Trypan blue method has the advantage over several classically used methods for comprehensive cell viability assessment, including the LDH assay because it is unaffected by cellular redox microenvironment (milieu) or redox chemical [38]. A concentration-dependent increase in trypan blue permeabilisation was observed (Fig. 3d), reflecting the increase in CHO-K1 cell death. A significant increase in the uptake of trypan blue (4, 16, and 31% at 5 μg/mL of MSN, MWCNT, and ZnO NPs, respectively) CHO-K1 cells was observed after 24 h of treatment. This increase in trypan blue uptake was further enhanced at 20 μg/mL and reached 3.5, 104% and 132% after 24 h of treatment (Additional file 1: Figure S2 and Additional file 2: Table S1).

The results prove that ZnO NPs have higher toxicity than MWCNT and MSN. 5 µg/mL dose of ZnO NPs selected as High Observed Adverse Effect Level (HOAEL) because, at this concentration, we found a 70% decrease in cell viability. Similarly, 5 µg/mL of MWCNT and 15 µg/mL of MSN doses were chosen as Low Observed Adverse Effect Level (LOAEL) because the concentration showed less than 20% decrease in cell viability. The comparative analysis of ZnO, MWCNT, and MSN cytotoxicity profile represented in hierarchical cluster-based on Pearson correlation (*P* < 0.05) in Additional file 1: Figure S2. Furthermore, the colony formation assay findings demonstrated the inhibitory effect of all three types of NPs (Fig. 3e). In contrast, no colonisation was observed after one week of assay upon the ZnO5 NPs exposure.

### Nanoparticles-associated changes in cell symmetry

Cell morphology was examined by both phase contrast (6, 12, and 24 h) and wright stain (24 h) (Fig. 1a, b). The results showed a significant decrease (1664 ± 451.7 μm^2^) in surface area on exposure with ZnO5 4259 ± 81.23 μm^2^ (*P* < 0.01) whereas, the surface area was significantly increased after exposure to MWCNT5 6491 ± 355.6 μm^2^ (*P* < 0.05). However, the MSN15 demonstrated no changes in surface area 4149 ± 465.8 μm^2^ after treatment (Fig. 1c). The initial aspect ratio (AR) of CHO-K1 cells was close to 2, but over 24 h exposure with ZnO5 decreased significantly around 1.55, which is higher than the MSN15 ~ 1.71 MWCNT5 ~ 1.69 (Fig. 1c). The circularity of all three NPs treated cells surfaces were observed dissimilar, but ZnO5 show the highest circularity ~ 0.53 compared to control ~ 0.31, whereas MSN15 show the lowest circularity ~ 0.26 (Fig. 1c). The typical CHO-K1 cell morphology is elongated and spindle-shaped, indicated by a high aspect ratio coupled with low circularity. MSN15 treated cells having high perimeter: area ratio, with low circularity, indicates no difference in the long and short axis of the control cells (Additional file 1: Figures S3–5 and Additional file 2: Tables S1.1–1.4).

In contrast, MWCNT5 treated cells appears more elongated or cuboidal shape showed by similar circularities and lower AR. On the other hand, cells treated with ZnO5 shifted towards the isotropic shape. In contrast, MSN15 and MWCNT5 showed anisotropic shape, whereas some of the cells underwent prominent protrusions. Analysis CHO-K1 cells using wright stain showed ZnO5 NPs exposure leads to cellular shrinking, nuclear condensation, and nuclear fragmentation followed by early apoptotic cell death. The exposure of MWCNT5 to CHO-K1 cells resulted in extensive cell swelling (cytoplasmic vacuolisation) and large surface. Chemically induced cytoplasmic vacuolisation of cultured cells usually harm the cell functioning. These cytoplasmic vacuoles may develop from different organelles like mitochondria, lysosome, and Golgi body [39]. Eventually, cellular destruction and death were observed with cellular vacuolisation progression, followed by intracellular content release [40]. Based on the above morphological alteration, we postulate that ZnO5 causes more significant cell damage than MWCNT5 and MSN15 in complementation with the colorimetric assay results. Cellular imaging by TEM analysis confirmed that more number of mitochondria present in MWCNT5 treated cells while ZnO5 treated cells showed early apoptosis with typical nuclear condensation and blebbing nuclear membrane (Fig. 2b). Potential cytotoxicity, along with extensive cell morphological alteration by these NPs, sparked our interest to explore the mechanism behind perturbations.

### Quantitative proteomics analysis of ZnO, MWCNT, and MSN exposed CHO-K1 cells

**Fig. 4:**
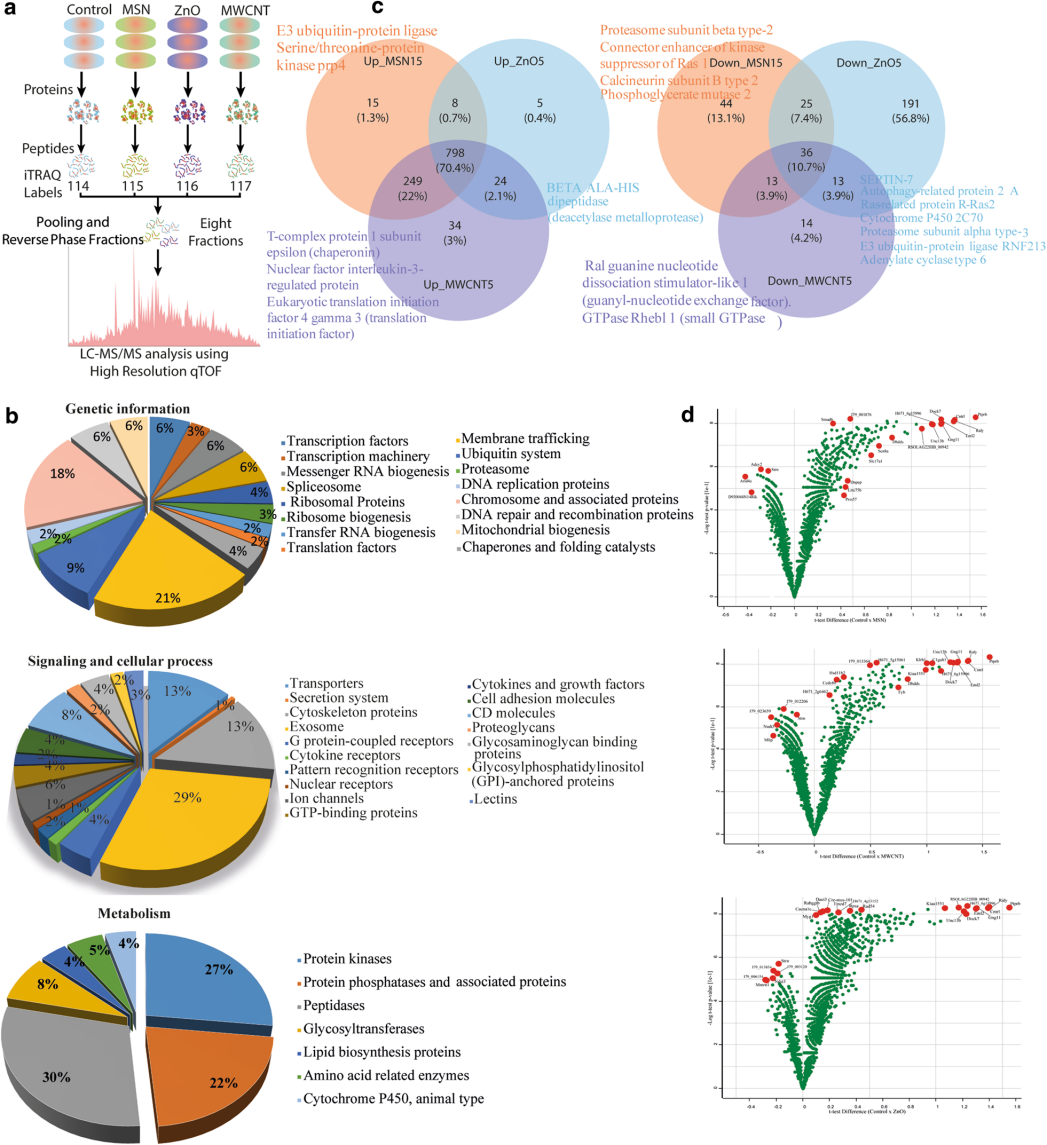

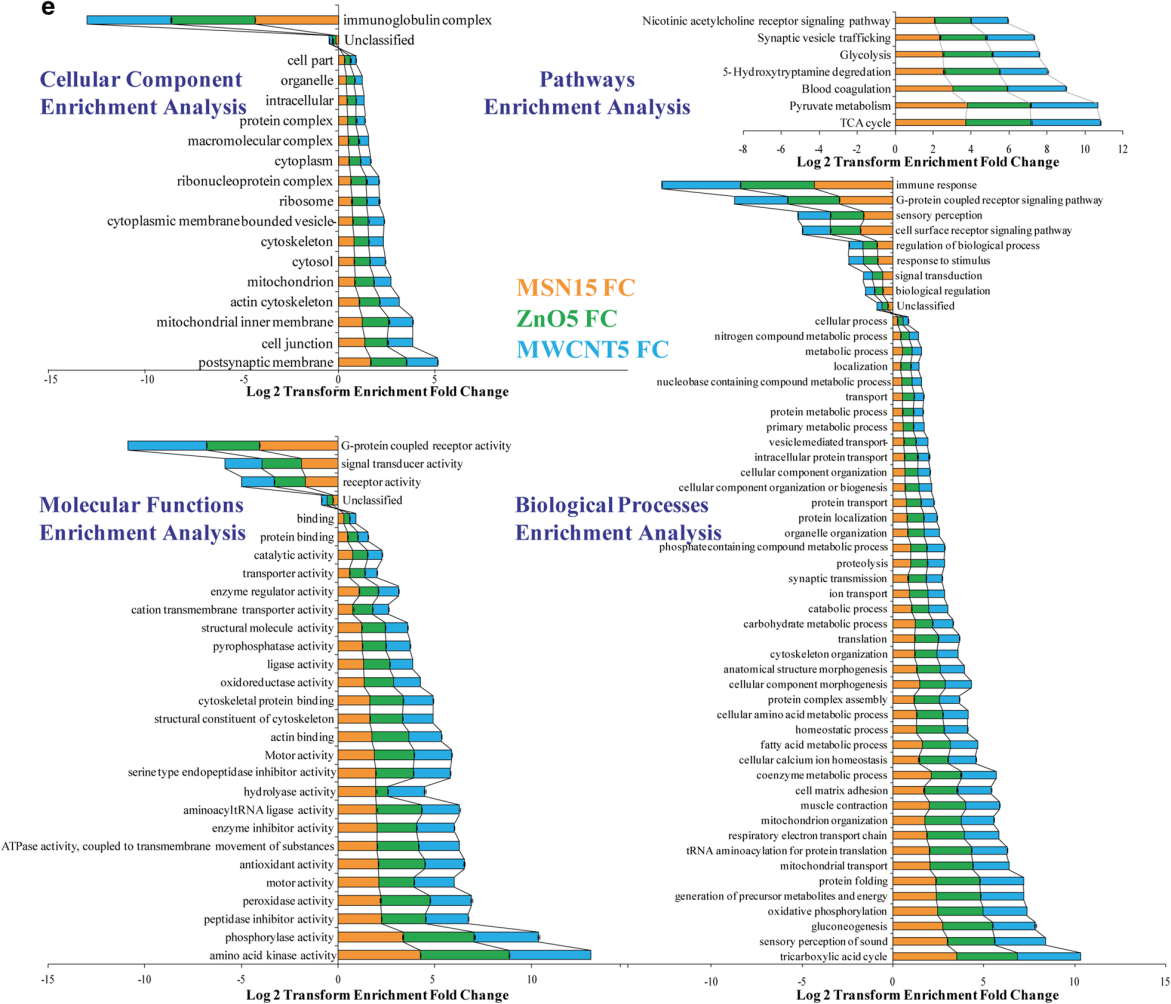
**a** Workflow for proteomic data analysis: (b) Graphical representation of all the normalised data obtained through Trans proteomics pipeline (TPP) tools. We have used three search engines commonly using the Uniprot *Cricetulus griseus* (Hamster), *Mus musculus* (Mouse) and *Rattus norvegicus* (Rat) database (Comet, Tandem, Mascot). Note: Control- Normal CHO-K1 cells, MSN- Mesoporous silica nanoparticle, ZnO- Zinc oxide nanoparticle, MWCNT- Multi-Walled Carbon Nanotube, Nanoparticle.114–117 are iTRAQ isobaric labels. **b** Functional classification differentially abundant proteins after MSN15, MWCNT5, and ZnO5 NPs treatment to CHO-K1 Cells: The pathways or bio-effects related to MSN15, MWCNT5, and ZnO5 NPs were determined by KEGG based annotation. The percentages of the differential proteins involved in the bio-effects of NPs were shown in the pie chart. 21% membrane trafficking, 18% chromosome, and associated protein, and 9% ubiquitin system classified to originate from genetic information. Signaling and cellular processes were identified 29% related to exosomal proteins, 13% cytoskeleton and transporter proteins. Metabolic associated proteins 30% assigned to the peptidases, 27% to the protein kinase, and 22% to the protein phosphatase of the total proteome. **c** Nanoparticles induces changes in the CHO-K1 Cell Proteome: In total, 6244 proteins were identified in NPs-treated and control CHO-K1 cells (peptide FDR p-value < 0.05). On fixed fold change cutoff at >|1.5|, total 1188 (19%) proteins were differentially expressed by the treatment of MSN15 (1070 upregulated and 118 down-regulated), 1181 (18.91%) proteins to MWCNT5 (1105 upregulated and 76 down-regulated) and 1100 (17.61%) proteins to ZnO5 NPs (835 upregulated and 265 down-regulated). Overlap among proteins up and down-regulated by MSN15, MWCNT5, and ZnO5 NPs. **d** Volcano plot elucidates significantly differentially abundant proteins in the CHO-K1 cell line after NPs treatment: The volcano plot displaying the fold changes (*x*-axis) *vs* the − log_10_ *p*-values (*y*-axis) for individual protein. An essential feature in the volcano plot selected by the *t*-statistics. The proteins are highlighted in each panel shows with larger and smaller fold changes (red). The upregulated proteins represented in the right side of the plot and the downregulated proteins represented in the left side of the plot. Note the values are on a log scale so that differential expression values can be plotted symmetrically. **e** Gene Ontology analysis: Gene Ontology (GO) analysis was performed of differentially regulated proteins by the MSN15, MWCNT5, and ZnO5 NPs. The bar graph indicate the upregulated and downregulated GO enrichment for the cellular component, pathway, molecular function and biological function of the proteins. The orange bars shows proteins enriched by MSN15 NPs, green bars show proteins enriched by MWCNT5, and sky blue bars show proteins enriched by ZnO5 NPs. The value of FDR < 0.05 was considered statistically significant

We carried out multiple assays with relevance to three types of NPs, and their specific proteomic responses suggested a clear vision of the adverse outcome pathway. The mass spectrometry dependent iTRAQ-based quantitative proteome analysis of the NPs-treated and control CHO-K1 cells were characterised (Additional file 1: Figures S6–7), and a total of 6244 proteins (peptide FDR *p*-value < 0.05) across all samples (Additional file 3: Table S2 and Additional file 4: Table S3). Initially, the KEGG based extensive annotation resulted in the 21% membrane trafficking, 18% chromosome, associated protein, and 9% ubiquitin proteins classified to originate from genetic information. While on associated metabolic proteins, 30% assigned to the peptidases, 27% to the protein kinase, and 22% to the protein phosphatase of the total proteome. Foremost signalling and cellular processes were identified 29% related to exosomal proteins, 13% cytoskeleton, and transporter proteins (Fig. 4b).

On fixed fold change cutoff at >|1.5|, total 1188 (19%) proteins were differentially expressed by the treatment of MSN15 (1070 upregulated and 118 down-regulated), 1181 (18.91%) proteins to MWCNT5 (1105 upregulated and 76 down-regulated) and 1100 (17.61%) proteins to ZnO5 NPs (835 upregulated and 265 down-regulated) (Additional file 1: Figures S6–7). The name of DEPs was presented in the Venn diagram (Fig. 4c), and the elaborated table is provided in Additional file 3: Table S2. The common proteins were involved in intracellular trafficking, proteasome, and ubiquitination complex were abundant in all NPs. We also observed upregulated proteins specific to MSN15, *i.e*., E3 ubiquitin-protein ligase rnf213, serine/threonine-protein kinase prp4 and pyruvate carboxylase. For example, T-complex protein one subunit epsilon (chaperonin), nuclear factor interleukin-3-regulated protein, eukaryotic translation initiation factor 4 gamma 3 (translation initiation factor), TP53-target gene five protein and proteasome subunit beta type were regulated explicitly by MWCNT5. Additionally, the ZnO5 NPs treatment-induced down-regulation of the several processes such as ubiquitination (Psma3, Ubl4, Pja1, Rnf40, and Rbbp6), cytoskeleton (Lasp1, St5, and Gas8), cell cycle (KIF14, Cenph, H2afx, and Zw10), DNA repair pathways (Rad54, Mlip, and Apex1) and cell proliferation (Ghitm, Olfm4, Efnb2, Rtn4, St5, and L1cam). Depletion of KIF14 impaired cell viability, anchorage-independent growth, cell cycle progression [41, 42], and induced apoptosis was down-regulated [Fold change (FC: 0.42)] in response to ZnO5 NPs.

The volcano plots (Fig. 4d) reflect the relative protein's richness in MSN15, ZnO5, and MWCNT5 treated cells compared with control (log_2_-ratio ≥ 0.2, p ≤ 0.05, t-test). The volcano plots for MSN15 v/s control (Fig. 4d1) and MWCNT5 v/s control (Fig. 4d2), are shifted slightly to the right, postulating that a high number of proteins are upregulated. In MSN15, the upregulated protein are PTPRB, dock7, Raly, Smad6, Cntrl and Unc13b, which increase the cell migration and invasion, and the down-regulated protein are STRN and ADCY2 that negatively regulates the cell proliferation. In case of ZnO5, the upregulated protein EML2, KIAA1551 and Rad54 involved in double-strand breaks (DSBs) repair, thereby resulting in prophase arrest due to unrepaired DSBs triggering the meiotic recombination checkpoint [43]. TMED7 and TMED10 (transmembrane emp24 protein transport domain containing 7 and 10) are essential for the trafficking of TLR4 from the ER to the cell surface by the Golgi [44]. In MSN15 and MWCNT5, both TMED7 and TMED10 are upregulated, which activate TLR4 [44]. TLR4 activation promotes acute and chronic inflammation.

### Nanoparticle specific changes in the CHO-K1 cellular proteome

Gene Ontology analysis was performed to gain depth into the cellular components, molecular functions, and biological process of these DEPs by the MSN15, MWCNT5, and ZnO5 NPs. The ZnO5 NPs (green bars) regulate more number of proteins than MWCNT5 NPs (Fig. 4e). Our results showed the enriched cellular component terms that are cell junction (*p* < 10^−4^), actin cytoskeleton (*p* < 10^−5^), cytoskeleton (*p* < 10^−5^), mitochondria (*p* < 10^−7^) and inner mitochondrial membrane proteins (*p* < 10^−4^). While in concordance with pathway regulated are TCA cycle (*p* < 10^−3^) and pyruvate metabolism (*p* < 10^−3^) to ensure the cell morphology and metabolic pathway disruption due to NPs exposure. Our results showed that all the processes majorly affected by all three NPs, like an increase in anti-oxidant (*p* < 10^−2^), peroxidase activity (*p* < 10^−2^), and a decrease in G-protein coupled receptor activity (*p* < 10^−4^). Additionally, when cells undergo oxidative stress, certain proteins are enriched (P4hb, MUS81, Il1r, and SOD1) that act as an anti-oxidant mechanism peroxiredoxin, which may counteract rapidly from stress. The release of hydrolases (*p* < 10^−2^) in the case of MWCNT5 and MSN15 high may activate the cathepsin B and D that is harmful to cells by initiating catholic degradation of cellular components, which possibly leads to apoptosis (Additional file 1: Figures S8–10).

**Fig. 5:**
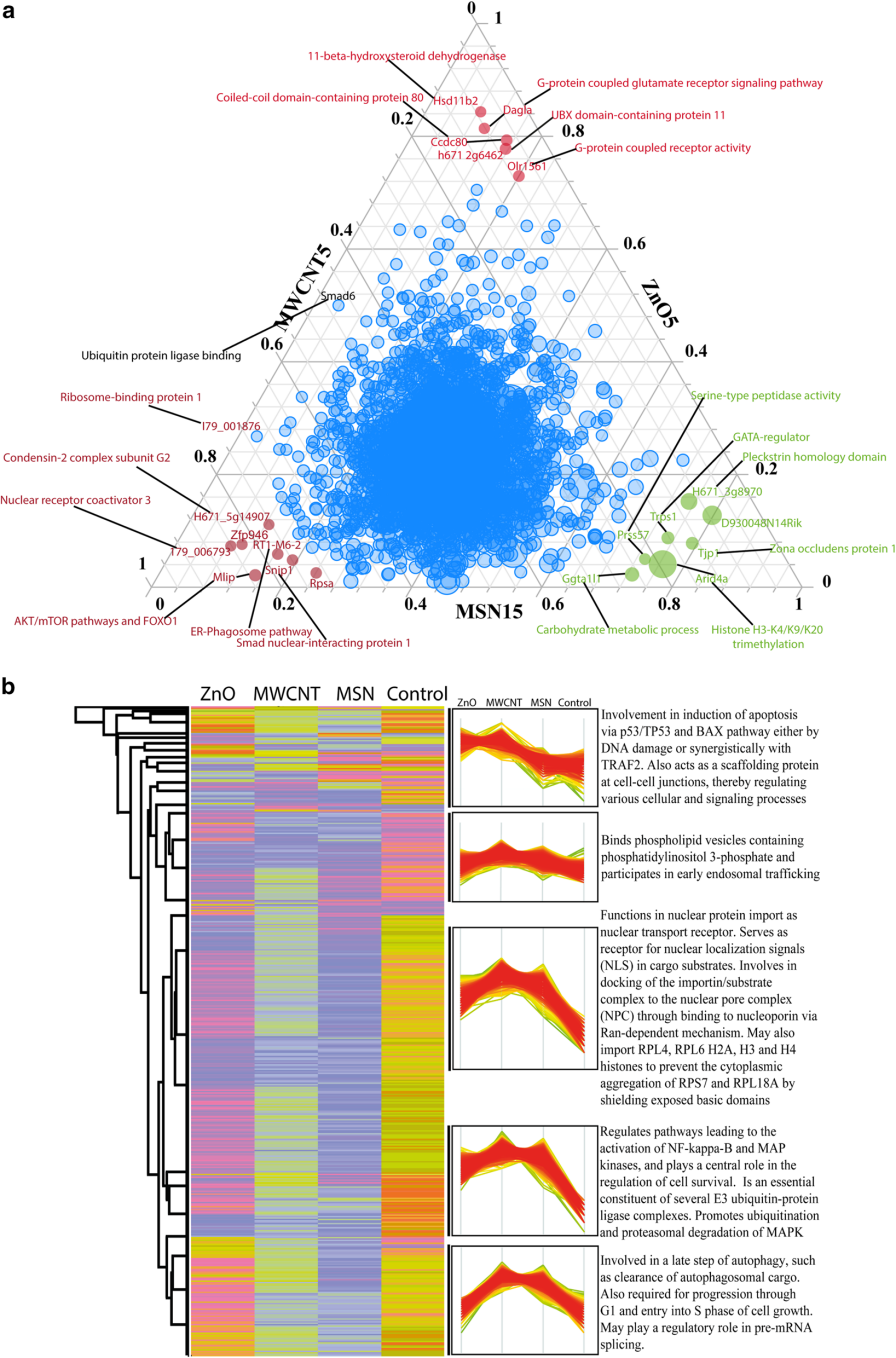
**a** Ternary plot: The distribution of proteins was observed in the center of the ternary plot under MSN15, MWCNT5, and ZnO5 nanoparticles, which indicate this phenomenon was global. GATA regulator, serine-type peptidase activity, carbohydrate metabolism, Zona-occludens protein 1, and histone H3-K4/K9/K20 trimethylation to be exclusively identified upon MSN15 stimulation. ER-phagosome pathway protein, AKT/mTOR pathway, Smad nuclear-interacting protein 1, condensin-2 complex G2 and nuclear coactivator 3 stimulated with MWCNT5. G-protein coupled receptor activity, UBX domain-containing protein 11 and 11-beta-hydroxysteroid dehydrogenase were regulated by ZnO5 nanoparticle. **b** Clustering of protein from control, MSN15, MWCNT5, and ZnO5 NPs sample: A heat map was created using three biological replicates of control, MSN15, MWCNT5 and ZnO5 NPs sample, and initially data were transformed into log10 value for normalisation. The hierarchical clustering were performed using Pearson's correlation. Each column corresponds a sample, and each row corresponds a protein. The row Z score of each protein was mapped in color (scaled expression value). The dark color denotes proteins that are higher in abundance, and light color denotes protein lower in abundance. The hierarchical matrix was divided into five main clusters in the form of a profile plot

**Fig. 6:**
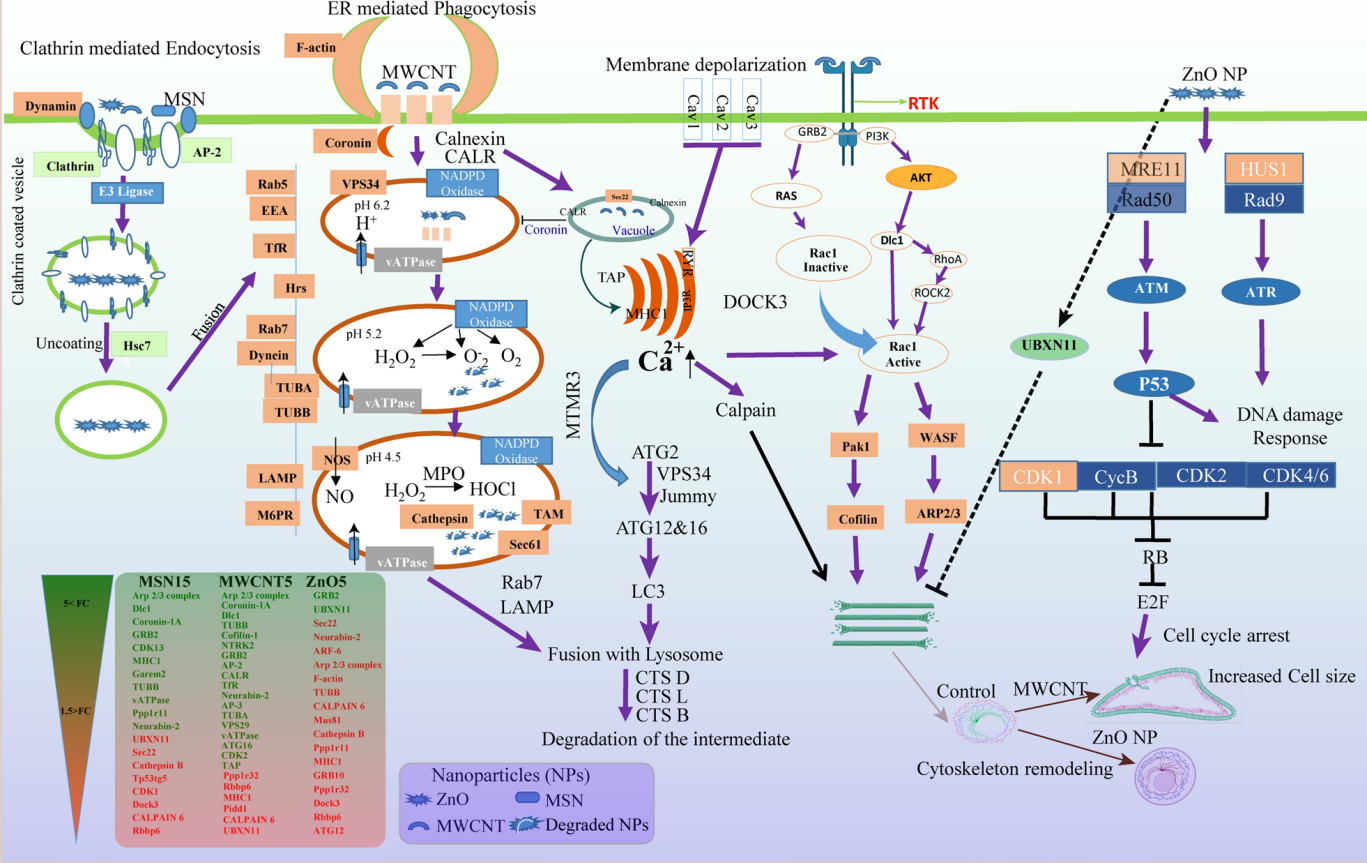
Schematic representation of the distinct pathway for internalisation of MSN, MWCNT, and ZnO NPs in CHO-K1 cells: The proposed mechanism involved MSN and ZnO NPs uptake through clathrin-mediated endocytosis. Phase 1, NPs binds with clathrin and adaptor proteins to form a clathrin-coated pit. The clathrin uncoated vesicles fused with early endosomes for subsequent endolysosomal trafficking. MWCNT internalised via ER-meditated phagocytosis involve the assembly of coronin, F-actin and other adaptor proteins on the plasma membrane for subsequent intracellular trafficking. ER play an essential role for recycling the receptor. Two distinct pathway regulate Rac1 pathway to modulate cytoskeleton dynamics or cell morphology. MWCNT synergise with other cell surface receptors like Cav to activate Ca^2+^ signaling through the ER. Ca^2+^ signaling linked to Rac1 pathway through DOCK3 protein. On the other hand, receptor tyrosine kinase (RTK) involved in Rho-Rac mediated signaling pathway to regulate the F-actin polymerisation. ZnO NPs upregulate the UBXN11 that induced the actin stress fiber formation and causes the cell rounding. The internalisation of ZnO nanoparticle can activate P^53^ signaling pathways and subsequently affect DNA damage repair mechanism and cell cycle arrest

Precisely, the comparative response for particular nanoparticles (ternary plot) (Fig. 5a) distinguished the differential proteins relative to one another. The distribution of proteins was observed in the centre of the ternary plot under MSN15, MWCNT5, and ZnO5 nanoparticles, which indicate this phenomenon was global. We found GATA regulator, serine-type peptidase activity, Zona occludens protein1 (ZO-1), and AT-rich interaction domain 4A (ARID4A) to be exclusively identified upon MSN15 stimulation. ZO-1 (FC: 5.3 and down-regulation in case of ZnO5 and MWCNT5) have a pivotal role for maintaining cell–cell adhesions, cell migration and cytoskeleton remodelling [45]. ARID4A (FC: 25 upregulated) is an essential regulator of cell growth and migration. ARID4A is retinoblastoma protein (pRB) binding protein that plays a vital role in tumour suppression and cell cycle arrest [46]. In 2006, Wu et al. found that ARID4A alter epigenetics modification at PWS/AS domain with increase methylation of histone H3-K4/K9/K20 that regulate genomic imprinting [47]. Our result suggests that MSN15 may be useful for developing potential therapeutic target in cancer. Furthermore, we found RT1 class I (MHC1), AKT/mTOR pathway, Smad nuclear-interacting protein 1, condensin-2 complex G2, and nuclear coactivator 3 when stimulated with MWCNT5 (Fig. 5a). ER-mediated phagocytosis required MHC class I and calnexin and calreticulin proteins [48] (detailed mechanism presented in Fig. 6). G-protein coupled receptor activity, UBX domain protein 11 (UBXN11), and 11-beta-hydroxysteroid dehydrogenase was regulated by ZnO5 nanoparticle (Fig. 5a). The upregulation of UBXN11 that is in the plasma membrane induced the actin stress fibres formation and cell rounding. Exposure to ZnO5 NPs induced the translocation of UBXN11 to the periphery to cell and led to the loss of adhesion. UBXN11 binds with Rnd GTPase causes the retraction of actin polymerisation in COS-7 cell [49].

### Comparing the proteome signatures after treatment ZnO5, MWCNT5, and MSN15

We investigate global proteomic differences and temporal responses after MSN15, MWCNT5, and ZnO5 NPs treatment; we employed unsupervised hierarchical clustering of the 6244 proteins with statistically different expression across all treatment (FDR < 0.05) (Additional file 4: Table S3). Numbers of proteins, fold change, selected enriched GO terms and KEGG pathways (Fisher's exact test, FDR 0.05) with their enrichment factors are presented for marked clusters (Fig. 5b). The heat map shows one significant cluster of highly and co-expressed proteins for each of the three treatments. The hierarchical matrix was divided into five central clusters in the form of a profile plot. Profile plot revealed that proteins in cluster 1 (highly expressed in the ZnO5 and MWCNT5) were enriched involvement in term induction of apoptosis via TP^53^ and Bax pathway either by DNA damage to MSN15 and control. ZnO5 increases the expression of following proteins that are p53-induced death domain-containing protein 1, UBX domain protein 6, IQ motif containing GTPase activating protein 1, lamin-A, and cell division cycle seven proteins. Those are known to induce apoptosis and catastrophic DNA damage. Cluster 2 (high expression in the MWCNT5 and MSN15, respectively) involved early endosomal trafficking (Table 1). The terms nuclear transport, ubiquitination, and proteasomal degradation were also enriched in this cluster 3, and 4 accordant with preventing the protein aggregation in the cytoplasm to play a central role in cell survival (Fig. 5b). MSN15 increases the expression of Clusterin, Prolyl 4-hydroxylase, beta polypeptide, Tripeptidyl peptidase II and Ubiquitin carboxy-terminal hydrolase L1, which function as chaperon to prevent the protein aggregation (Additional file 1: Figures S8–14).

**Table 1:** Proteins involved in an important biological process found to be differentially regulated by MSN15, MWCNT5 and ZnO5 nanoparticles

Biological Process	Protein name	MSN15/control	ZnO5/control	MWCNT5/control
Clathrin mediated Endocytosis	SRP-9 Snap91, AP-2 Clathrin heavy chain 1 Siglec-1 Dynamin ARF-6 Arp2/3	2.88 3.33 4.28 2.9 2.63 2.9 2.23 2.27	4.33 2.44 2.28 1.8 1.72 2.36 1.30 2.36	2.88 3.88 6.42 4.1 3.72 2.72 3.15 3.09
ER-mediated Phagocytosis	Coronin-1A F-actin Sec22 MHC1 TAP CALR Calnexin	9.5 2.16 1.26 1.5 2.75 4.5 2.7	2 1.16 1.4 0.68 3.62 2.14 1.9	11.5 3.16 2.46 1.36 5.1 6.4 4.1
Early Endosome	Rab7 TfR vATPase TAP CALR Calnexin AP-2	4.5 4.7 5.71 2.75 4.5 2.7 2.8	2.87 2.42 2.41 3.62 2.14 1.9 2.1	4.12 6 5.28 5.1 6.4 4.1 3.9
Late Endosome	Rab5 TUBA TUBB Stx7 AP-3	2.45 3.75 5.83 1.54 3.6	1.81 1.75 1.16 2.09 2.1	3.45 5.37 8 4.36 5.8
Lysosome	LAMP Cathepsin M6PR	2.63 3 3.11	1.90 1.8 3	3.36 4.1 4
Autophagy	VPS29 Cathepsin D Cathepsin B LAMP ATG12 ATG16 Frizzled-8	4 3 1.22 3.77 1.64 3.87 2.6	2 1.8 0.77 1.88 0.35 2.25 1.27	5.3 4.1 2.17 4.33 2.88 5.25 3.18
DNA Damage and Repair associated proteins	Brat1 ATR protein kinase Tp53 Pidd1 Tp53i13 Tp53tg5 CDK1 CDK2 CDK13 Rbbp6 Garem2 GRB10 GRB2 Mus81 Igfbp3	3.36 2.54 2 1.56 4 1.22 1.21 4.42 7 0.69 5.85 4.22 8.5 1.61 2.88	1.36 2.09 2.4 2.31 3.25 1.5 2.5 2.57 3.33 0.5 2 0.66 8.5 0.83 2.33	3.36 3.45 4.6 1.31 4.25 1.77 2.5 5.17 4.16 1.38 5.28 5.22 7 2.11 4.55
Cytoskeleton Network Modulator	CALPAIN-2 CALPAIN-10 CALPAIN-6 CALPAIN-11 Dlc1 Dock3 Rac1 Pak1 Cofilin-1 Dynamin-1 Arp 2/3 complex Coronin-1A Rock2 UBXN11 CMFR1 Neurabin-2 Plexin-B1 WASF CDC42bpd Ppp1r11 Ppp1r32 NTRK2 Alpha-actinin-4	2 2.9 0.92 3.1 10.25 1 3 3.4 4.33 1.69 10.5 9.5 2 1.4 2.2 5 3.11 2.6 4.85 5.2 2.1 4.83 3.22	2.27 1.8 0.84 1.5 2.75 0.53 2.66 1.7 3.33 2 1.25 2 2.63 7 2.8 1.42 2.77 1.9 2.71 0.71 0.63 3.5 1.5	3.54 4.3 1.11 4.2 11 4.06 4 3.6 8 3.07 12.25 11.75 3.45 0.6 2.9 5.85 4.2 4.2 4.71 7.14 1.5 7.3 4.5

Furthermore, it helps in lysosomal and proteasomal degradation of MSN nanoparticle degradation and cell proliferation. Our study suggests that targeting the ubiquitin system with MSN15 can offer a new therapeutic approach for treating cancer, infectious diseases and neurodegenerative diseases. The enrichment of autophagy such as clearance of autophagosomal cargo and cell growth regulation through G1 and entry into S phase observed in cluster 5 (high expression in the MWCNT5 and MSN15, respectively). MWCNT5 upregulated proteins, calreticulin, lamin-A/C, and Rap1 interacting factor 1, indicating that MWCNT5 exposure induced the calcium-mediated signalling cascades in a cell (Additional file 1: Figures S11–14). Lamin-A/C and Rap1 arrest the mitosis and S-phase of the cell cycle, respectively, which leads to genomic instability and premature cell senescence. Our result is similar to the previous finding that silver NPs induced DNA damage, apoptosis, and ubiquitination of protein in LoVo cells [50]. We found that number of proteins related with ubiquitination, proteasome, and DNA damage repair were upregulated upon the exposure of MSN15, MWCNT5, and ZnO5 (Table 1), showed that proteins damaged by the nanoparticles go to degradation via ubiquitin–proteasome complex and replaced by de-novo synthesis. In general, ubiquitination plays an essential role in the exposure of nanoparticles to the human cell. In 2013, Yan and collaborators observed the cellular uptake of nanoporous polymer particles (NPPs), regulate the ubiquitination, which can be castoff as drug delivery systems [51].

### Cell signalling initiated through ZnO5, MWCNT5, and MSN15

**Fig. 7:**
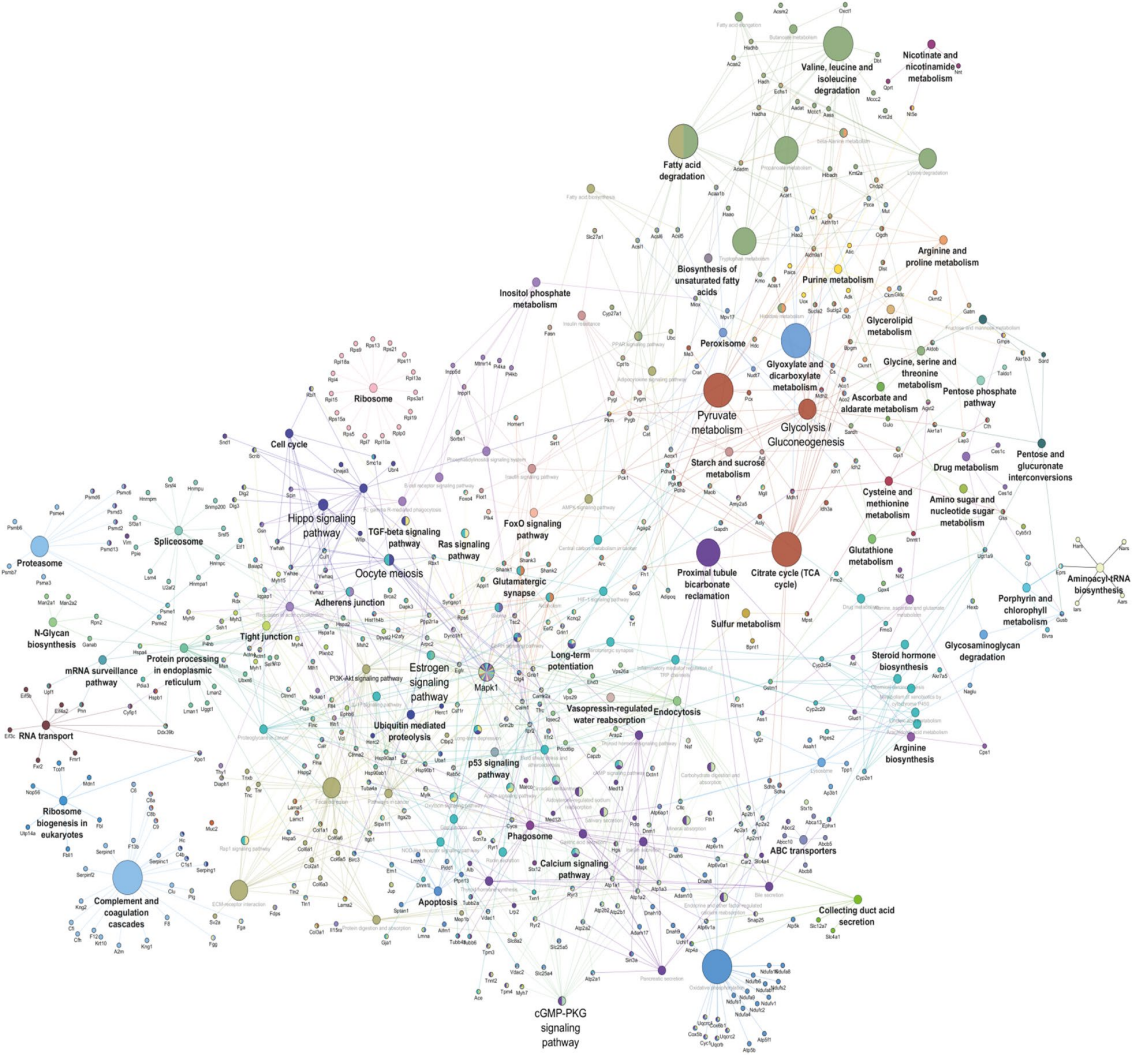
Functionally grouped network of enriched biological pathways: The proteins networks were generated with high confidence interaction (overall 0.90 scores) in Cytoscape platform together in combination with Reactome database. Using the kappa score based statistics model, it provides the degree of relationship among the pathways associated with significant relevant overlapping proteins and Gene Ontology (GO) terms. The threshold 0.4 Kappa statistics was set, and above that, all the terms connections were selected to illustrate the significant interactome. The analysis uses the following parameters minimum three and maximum eight-level for GO tree, minimum 3% of genes of GO term/pathway, Benjamini and Hochberg test with the two-sided hypergeometric distribution of corrected FDR level < 0.05 significance. The protein–protein interaction maps represents the Akt/mTOR pathway, Map/Erk pathway, DNA damage responsive pathway and Ca^2+^ signaling cascade and endolysosomal machinery that include ubiquitination and proteasomal degradation

The analysis demonstrated the response initiated by each NPs differentially regulates downstream signalling pathways. Moreover, the results were confirmed with protein–protein interaction (PPI) maps; consequently, we observed ablation in endolysosomal machinery that includes ubiquitination and proteasomal degradation. Furthermore, there is a close association with DNA damage repair pathway and apoptosis. The cluster associated with ubiquitination, including Uba family proteins and Usp family proteins, is linked to proteasomal degradation, including Psme, Psmc, and Psmd family proteins. We also observed apoptosis signalling, including protein, Bad, Ndufs family proteins, Uqcrc1, Uqcrc2 and calreticulin, is the connection among the cluster related ubiquitination and proteasomal degradation to the cluster linked to apoptotic signalling pathway. Hence, the PPI analysis confirmed the role of endoplasmic reticulum and mitochondria in nanoparticle-induced stress. However, other signalling pathways were affected mainly by MSN15, MWCNT5, and ZnO5, as prominent activation of the Akt/mTOR pathway, Map/Erk pathway, DNA damage responsive pathway and Ca^2+^ signalling cascade (Fig. 7).

In regards to all three NPs, we found that it enters the CHO-K1 primarily through clathrin-mediated pathways, ER-mediated phagocytosis, and get stored in endocytic compartments (Additional file 1: Figures S11–14). Notably, the proteomic finding revealed that same set the endocytic proteins upregulated (Table 1) and thus increase the subcellular space available for nanoparticle-corona complex. The protein set engaged with clathrin-mediated endocytosis, including SRP-9 (about three-fold abundant) protein required for early formation of clathrin-coated pits (Snap91, AP-2, and Clathrin heavy chain 1: 2.5 fold upregulated). Siglec-1 binds to membranes highly enriched in phosphatidylinositol 4,5-bisphosphate and promotes membrane tabulation. Our proteomic analysis revealed several ER resident proteins involved in the MWCNT5 uptake (ER-mediated phagocytosis model represented in Fig. 6). According to our model, MWCNT5 contact with cell and cell formed pseudopodia for trapping (Fig. 2b) after that ER recruited at the MWCNT5 contact site and fused with the plasma membrane, which induced the pseudopodia elongation and actin cytoskeleton remodelling. As an earlier finding shows that ER resident proteins calnexin and calreticulin are involved in phagocytic cups formation [52].

Interestingly, our data showed upregulation of calreticulin (FC: 6.4) and calnexin (FC: 4.1) and these finding indicate that ER proteins involved in the phagocytosis process. Furthermore, sthe involvement of SEC22 (FC: 2.46) and F-actin (FC: 3.16), which regulate the membrane fusion and cytoskeleton organisation, can support the ER's role in forming the phagosome. While each NPs has a unique set of secreted proteins and likely modulate the different cell manners. Initially, crescent shape vacuole was found by TEM studies in case of MWCNT5 exposure (Fig. 2b) as the earlier report suggests that ER-mediated phagocytosis also associated with reduced the endolysosomal fusion compartment and inhibit the phagosome maturation [53]. We suggested that MWCNT-derived vacuole localised proteins syntaxin 2 & 3 and SNAP23 interacts with the ER-localised protein Rab1A (FC: 3.42) and Sec22b (FC: 2.47). The previous finding indicates intracellular bacterial pathogen *Legionella pneumophila* induced plasma membrane syntaxins and Sec22b proteins interaction [54]. Future study will shed light towards how the fusion of MWCNT‐derived vesicles with ER regulates the phagosome maturation. Our study could provide important insight towards development nanomedicine for organelle-specific targeting.

Additionally, local and global calcium signals regulate ER-mediated phagocytosis, which controls the different steps of endolysosomal fusion and cytoskeleton dynamics. In comparison, localised signals generated from caveolin-1 (FC: 3.45) induced the ER-phagosome complex formation and generated the calcium hotspot in the cytosol. On the other hand, global signals were linked to the phosphatidylinositol 3-phosphate (PtdIns3P) release regulating the Rho kinase-dependent MLC phosphorylation to modulate the cytoskeleton organisation and cell morphology (detailed mechanism in Fig. 6). In 2017, Li, H and coworker suggested that carboxylated multiwalled carbon nanotubes (c-MWCNTs) increase migration of RAW264.7 cells by stimulating the IP3/PLC/CRAC channel signalling pathway [55]. As previous reports suggest, gelsolin and ADF/cofilin play complementary roles to control the recycling of actin cytoskeleton regulated by calcium and phosphoinositides [56].

Further, the up-regulation of gelsolin (MWCNT FC: 4.44 and MSN FC: 2.89) in our study provide additional clues towards actin cytoskeleton required for phagocytosis, cell migration and enlargement. Rab5 and -7, ATG12, and LAMP1 that triggers endocytosis and autophagosome complex formation. It provides a protected environment for the release of the NPs to the sites. Altogether, our finding suggests a mechanism whereby the endolysosomal pathway is coupled for all three types of NPs, and this same pathway may provide a means for its elimination through ubiquitination and proteasomal degradation.

On the other hand, ZnO5 has shown direct, and clathrin-mediated trafficking behaviour since a low level of up-regulation endolysosomal proteins were found. The previous study suggests that ZnO NPs direct transport to intracellular locations and stable in the intracellular niche for release of ion to cause toxicity [57]. Similarly, our results confirmed that after endocytosis ZnO5 NPs travels through the endolysosomal compartment to reach the nucleus and initiate DNA break, clearly indicate that endosomal proteins play a crucial role in the DNA damage. Our TEM result showed a higher accumulation of ZnO1 and ZnO5 NPs in the nucleus. In 2015, Heim, J, and colleagues also observed that ZnO NPs have a higher level in the nucleus than the cytoplasm and induced DNA double-strand breaks [58]. In this context, we observed a higher level of cancer-associated gene-one protein, DNA repair protein Mus81, p53-induced death domain-containing protein 1, Serine/threonine-protein kinase ATR, Brca2, Paternally-expressed gene three protein, Striatin, MutS2 this an essential candidate of proteins for maintaining DNA-integrity and genomic stability.

### Effect of ZnO, MWCNT, and MSN on CHO-K1 cell migration

**Fig. 8:**
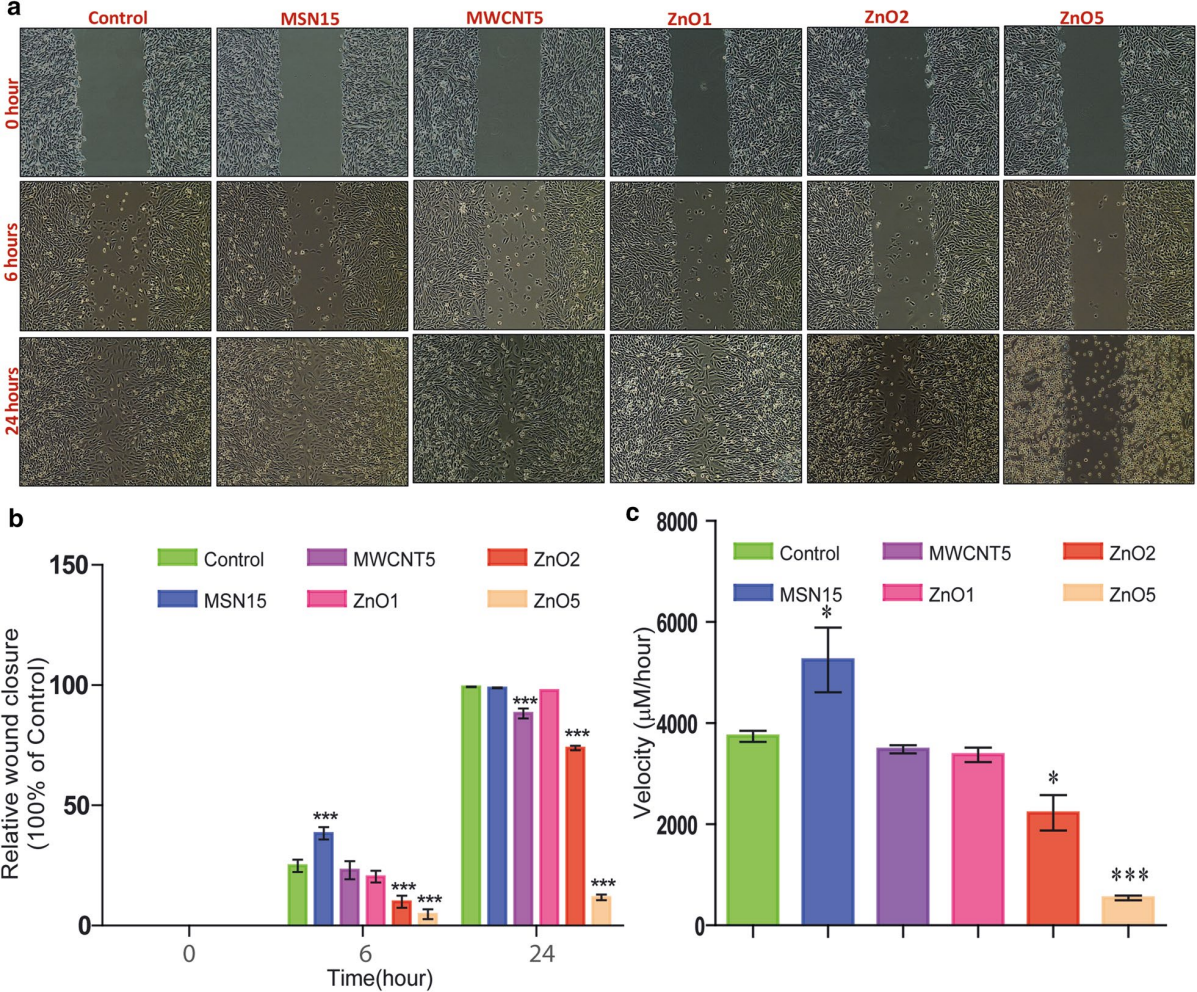
**a** Effect of nanoparticles on cell migration of CHO-K1 cells: CHO-K1 cells were cultured in 6-well plates and wounding on the after 48 h. CHO-K1 cells treated with MSN15 NPs (5 µg/mL) MWCNT5 (5 µg/mL), ZnO1, ZnO2, ZnO5 NPs (1, 2 and 5 µg/mL) and control cells. The images were captured at hour 0, 6, and 24 h, respectively, after the wound was created. **b** The results are expressed as relative wound closure and velocity (mean ± SD) were calculated by one-way ANOVA with Tukey's posthoc test (*p < 0.05, **p < 0.01,***p < 0.001) from three independent experiment

One of the most critical cellular parameters that readily affected by nanoparticles is the migratory ability of cells (Fig. 8a). The migratory potential also assessed by wound healing assay on CHO-K1 cells after treatment with the ZnO5, MWCNT5, and MSN15 NPs. Compared to control, MWCNT5, ZnO1, and ZnO5 showed a significantly reduced percentage of wound closure (Fig. 8b) over the assay duration. In the case of MSN15 treatment increases the migration of cells ~ 53.95% after six hours of treatment with rate 5248 ± 639 μm/h as compared to control 3741 ± 106 μm/h (p < 0.05). Six-hours after exposure, MWCNT5 and ZnO5 lead to decreased migration to 7.27 and 81.07%, at a rate of 3484 ± 81.67 545.7 ± 47.42 μm/h, respectively (p < 0.001). Thus, the ZnO2 and ZnO5 treated cells fail to close the wound after 24 h of scratching of the monolayer, proving that the cell migration was retarded significantly after ZnO NPs treatment. The lowest concentration of ZnO was 1 µg/mL, which was deliberately efficient to decrease migration ability cells. As per our expectation, we found significantly increased migration upon MSN15 treatment. The significant inhibition in migration was observed in ZnO5, and MWCNT5 treatment, hence the gap tempted in the wound assay was sustained.

The previous study reported that MWCNTs produce centrosomal mispositioning and disrupt the nuclear-centrosomal axis hindering cell migration [12–14]. The same results were confirmed using various cancer cell lines (HeLa cells, MCF7 cells, SH-SY5Y cells, and U87MG cells) incubated with 25 μg/mL MWCNTs for 70 h. This MWCNT concentration produced no apparent toxicity signs but revealed a statistically significant cellular migration speed reduction [14]. Cell migration processes are controlled by the reorganisation of the actin cytoskeleton, which is regulated mainly via Rho and Rac GTPase subfamilies that play an essential role in adapting the cell to different microenvironment [59]. The cells ability to adhere to the other cells or extracellular matrix is tightly regulated through survival and apoptosis signals [60, 61].

In most cases, this is operated by integrin-mediated FAK [62]. Results with both MWCNT5 and MSN15 showed an increase in calpain activity and required cleavage of FAK, which promotes cell spreading and enhances migration [63]. Dlc-1 members of the Rho family of small GTPases have an essential role in cell polarity, cell adhesion, and cytoskeletal organisation highly upregulated (about 10.25 fold) in case of MSN15 and MWCNT5. Kim, T.Y, and coworker (2009) suggested that overexpression of Dlc-1 enhances the migration but slows down the directionality to reflect variations in cytoskeletal dynamics and focal adhesions disruption random extension of long protrusions [64]. We also found decreases in migration and diminished directionality, which could be regulated by Dlc-1 silencing of CDC42BPA (about 0.54 down-regulated). The previous study suggests that active Rac1 (about four-fold abundant in MWCNT5) is essential for protrusive edges formation and cell polarisation [59].

Soenen, S.J and coworker (2010) reported that high intracellular iron oxide nanoparticle concentrations affect cell polarisation in a concentration-dependent manner. High levels of iron oxide affect the actin cytoskeleton and the formation and maturation of focal adhesion complexes (FACs) and can affect protein expression levels and could have detrimental effects on cell migration and differentiation [65]. Interestingly, a critical candidate regulating actin dynamics, cell migration, and membrane protrusions such as cofilin has been found eight-fold upregulated in case MWCNT5 exposure. Cofilin activity regulated through downstream of Rac1 via interaction with Pak1 (3.5 fold upregulated) to control the membrane protrusions by actin networks [66]. Our results from TEM and morphological analysis showed that MWCNT might provide a 3D microenvironment; the protrusive force of actin polymerisation drives cells into an elongated cellular morphology, which is sufficient for increasing ECM alignment. MWCNT acts as a scaffold for spermatogonial cell and maintains their proper shape and adherence [67] and supporting the use of MWCNT as a scaffold in tissue engineering. Rounded cell morphology is linked with increased MLC2 phosphorylation and actomyosin contractility. It was pointed out that inhibition of DOCK3 induced MLC2 phosphorylation, thereby actomyosin contractility [68]. Our results showed that DOCK3 down-regulated (FC: 0.53) in the case of ZnO5 and 4.06 fold abundant in MWCNT5. Our finding suggested that cell roundness can be regulated by two distinct pathway; UBXN11 protein regulates the assembly of actin stress fibres and cell–matrix adhesion. On the other hand, DOCK3 protein involved in transient expression of Rho-Rac signalling pathway.

Further studies are required for the identification of UBXN11 and other proteins involved in cell polarisation. In MSN15, we observed enhanced cell migration likely due to adaptation to stress or better delivery of nutrients. In addition to size, concentration and composition of the NPs contribute crucially to particles behavioural characteristics in the biological systems.

## Conclusion

The current study reports that NPs interactions with cells can alter biophysical parameters (morphological, cell spreading and migration) in correction with the cytoskeleton network's involvement. Changes expression levels of Dlc-1, UBXN11, DOCK3, and Rac1 are essential to control their morphology, proliferation, and migration in different type of NPs microenvironments. Notably, ZnO NPs induced stress fibre formation, which is linked with plasma membrane blebbing, and differential regulation in the level of UBXN11 and DOCK3 proteins. MWCNT promote adhesive phenotype as reflected by the Rac1 GTPase activation via complex, accompanying Dlc-1 and DOCK3. On the contrary, MSN did not alter cell proliferation and morphology, even though aggregated in the cytoplasm and might enter the endolysosomal compartment without invoking cytotoxic effects. Our study elucidates the molecular transport mechanisms that will provide a new perspective on designing nanoparticles as drug delivery systems.

## Supplementary Information

**Additional file 1: Figure S1.** Magnified image of TEM analysis. **Figure S2.** Heatmap of comparative cytotoxic response of nanomaterials: The heatmaps show the hierarchical clustering analysis of LDH assay, Trypan blue, MTT and WST-8 assay in CHO-K1 cells exposed to mesoporous silica nanoparticles (MSN), multiwalled carbon nanotube (MWCNT) and zinc oxide (ZnO) nanoparticles with different concentration of NMs. Highest cytotoxicity indicated red color whereas lowest cytotoxicity represented in blue color. **Figure S3.** Morphological changes in CHO-K1 cells under bright field microscope. A. 100X magnification. **Figure S4.** Morphological changes in CHO-K1 cells under bright field microscope. Effect of nanomaterials on cell morphology of CHO-K1 cells after MSN, MWCNT and ZnO NPs treatment. CHO-K1 cells were seeded in 6-well plates and nanomaterials for 24 hours for the stabilization of cells. CHO-K1 cells treated with MSN (15 and 50 μg/ml), MWCNT (5 and 20 μg/ml) and ZnO NPs (1, 2 and 5 μg/ml) and control cells for 24 h. Photographs were taken after 24 h, stain with Wright stain. 400X magnification. **Figure S5.** Morphological changes of CHO-K1 cells analysed at 24 h post-treatment of MSN, MWCNT and ZnO NPs. (A) Area, (B) Aspect Ratio (AR), (C) Circularity and (D) Roundness measurements were taken. **Figure S6.** Histogram analysis: Total intensity count for the reporter ions identified per channel labelled. 114 label for the control, 115 is MSN treated, 116 is ZnO treated, and 117 is MWCNT treated. All the protein identified in the channel followed the normal distribution. Each bin the histogram represent total number of counts for log2 transformed intensities. **Figure S7.** Multi Scatter plot analysis: All the identified reporter intensities were compared for the correlation among each other. The data represent high correlation among the nano-particle treatment. All the Pearson comparison values were represented in the left top corner of the scatter plot. **Figure S8.** Gene Ontology analysis: Only differentially regulated proteins were subject to Gene Ontology (GO) analysis. These pictures show the up- and down regulated GO terms for biological processes of the proteins. The blue bars represent proteins regulated by MSN NPs, red bars represent proteins regulated by MWCNT and the green bars represent proteins regulated by ZnO NPs. **Figure S9.** Gene Ontology analysis: Only differentially regulated proteins were subject to Gene Ontology (GO) analysis. These pictures show the up- and down-regulated GO terms for molecular function and cellular processes of the proteins. The blue bars represent proteins regulated by MSN NPs, red bars represent proteins regulated by MWCNT and the green bars represent proteins regulated by ZnO NPs. **Figure S10.** Gene Ontology analysis: Only differentially regulated proteins were subject to Gene Ontology (GO) analysis. These pictures show the up- and down-regulated GO terms for protein class of the proteins. The blue bars represent proteins regulated by MSN NPs, red bars represent proteins regulated by MWCNT and the green bars represent proteins regulated by ZnO NPs. **Figure S11.** The phagosome KEGG pathway: Green rectangle means the identified proteins and white rectangle means reference pathway. **Figure S12.** The endocytosis KEGG pathway: Green rectangle means the identified proteins and white rectangle means reference pathway. **Figure S13.** The Rap1 KEGG pathway: Green rectangle means the identified proteins and white rectangle means reference pathway. **Figure S14.** The cellular senescence KEGG pathway: Green rectangle means the identified proteins and white rectangle means reference pathway.

**Additional file 2: Table S1.1.** Cytotoxicity of MSN, MWCNT and ZnO NPs in terms of mitochondrial activity by MTT and WST-8 assay. **Table S1.2.** Cytotoxicity of MSN, MWCNT and ZnO NPs in terms of membrane damage by LDH release assay. **Table S1.3.** Cytotoxicity of MSN, MWCNT and ZnO NPs in terms of viability by Trypan blue uptake assay. **Table S1.4.** Cytotoxicity of MSN, MWCNT and ZnO NPs in terms of morphology altered.

**Additional file 3: Table S2.** Comparative proteomic profile of MSN15, ZnO5 and MWCNT5 NPs.

**Additional file 4: Table S3.** Gene Ontology analysis in response to MSN15, ZnO5 and MWCNT5 NPs: Only differentially regulated proteins were subject to Gene Ontology (GO) analysis. These Table show the up- and down-regulated GO terms for cellular component, biological process, molecular function and pathway of the proteins.
