# Supplementary material for: MSN, MWCNT and ZnO nanoparticle-induced CHO-K1 cell polarisation is linked to cytoskeleton ablation
Source: J Nanobiotechnology. 2021 Feb 12;19:45. doi: 10.1186/s12951-021-00779-7 (PMC7881565; doi:10.1186/s12951-021-00779-7)
Supplement: Supplementary file 1 — Additional file 1: Figure S1. Magnified image of TEM analysis. Figure S2. Heatmap of comparative cytotoxic response of nanomaterials: The heatmaps show the hierarchical clustering analysis of LDH assay, Trypan blue, MTT and WST-8 assay in CHO-K1 cells exposed to mesoporous silica nanoparticles (MSN), multiwalled carbon nanotube (MWCNT) and zinc oxide (ZnO) nanoparticles with different concentration of NMs. Highest cytotoxicity indicated red color whereas lowest cytotoxicity represented in blue color. Figure S3. Morphological changes in CHO-K1 cells under bright field microscope. A. 100X magnification. Figure S4. Morphological changes in CHO-K1 cells under bright field microscope. Effect of nanomaterials on cell morphology of CHO-K1 cells after MSN, MWCNT and ZnO NPs treatment. CHO-K1 cells were seeded in 6-well plates and nanomaterials for 24 hours for the stabilization of cells. CHO-K1 cells treated with MSN (15 and 50 μg/ml), MWCNT (5 and 20 μg/ml) and ZnO NPs (1, 2 and 5 μg/ml) and control cells for 24 h. Photographs were taken after 24 h, stain with Wright stain. 400X magnification. Figure S5. Morphological changes of CHO-K1 cells analysed at 24 h post-treatment of MSN, MWCNT and ZnO NPs. (A) Area, (B) Aspect Ratio (AR), (C) Circularity and (D) Roundness measurements were taken. Figure S6. Histogram analysis: Total intensity count for the reporter ions identified per channel labelled. 114 label for the control, 115 is MSN treated, 116 is ZnO treated, and 117 is MWCNT treated. All the protein identified in the channel followed the normal distribution. Each bin the histogram represent total number of counts for log2 transformed intensities. Figure S7. Multi Scatter plot analysis: All the identified reporter intensities were compared for the correlation among each other. The data represent high correlation among the nano-particle treatment. All the Pearson comparison values were represented in the left top corner of the scatter plot. Figure S8. Gene Ontology analys [file 12951_2021_779_MOESM1_ESM.pdf]

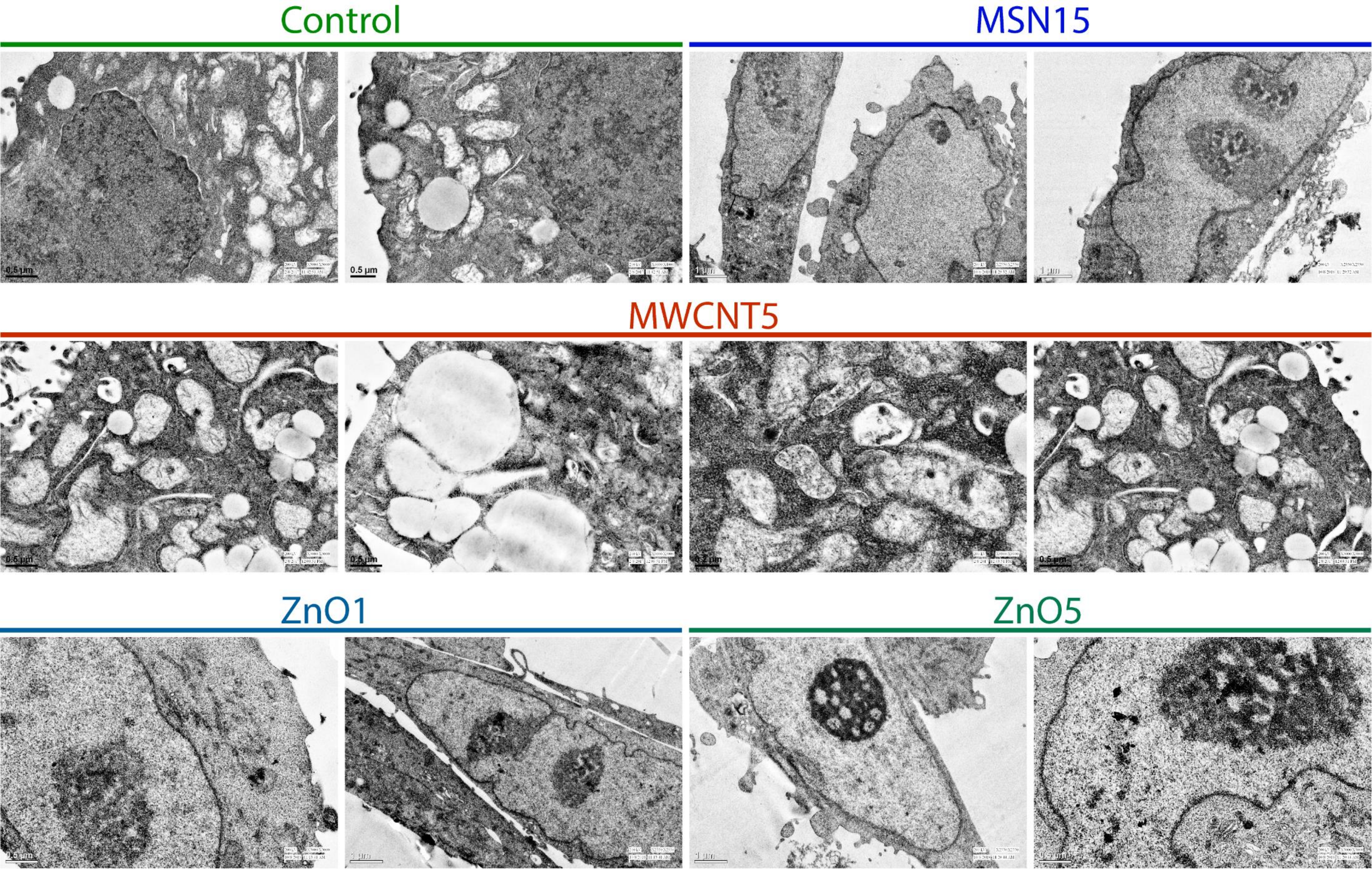

Figure S1: Magnified image of TEM analysis.

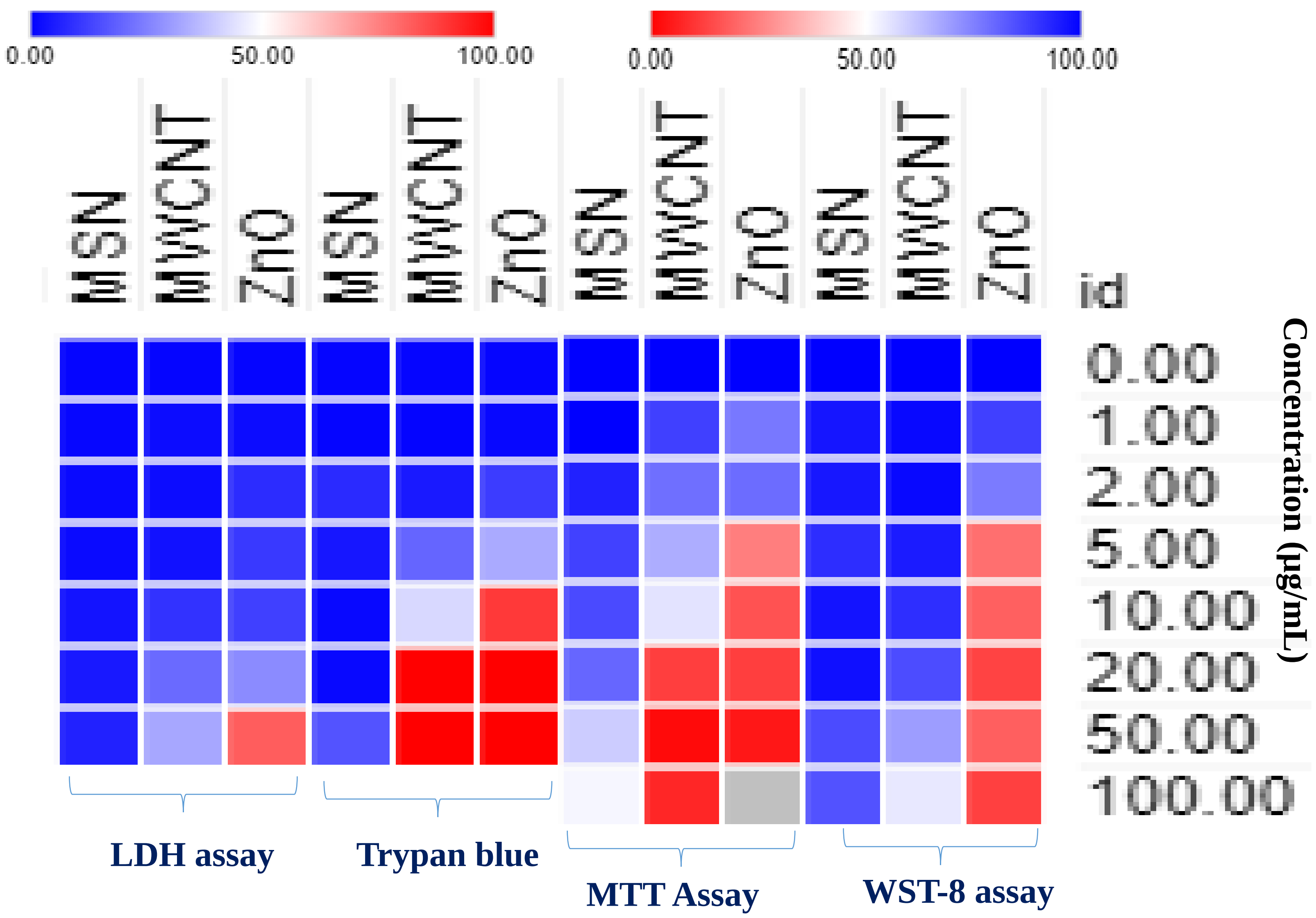

Figure S2: Heatmap of comparative cytotoxic response of nanomaterials: The heatmaps show the hierarchical clustering analysis of LDH assay, Trypan blue, MTT and WST-8 assay in CHO-K1 cells exposed to mesoporous silica nanoparticles (MSN), multiwalled carbon nanotube (MWCNT) and zinc oxide (ZnO) nanoparticles with different concentration of NMs. Highest cytotoxicity indicated red color whereas lowest cytotoxicity represented in blue color.

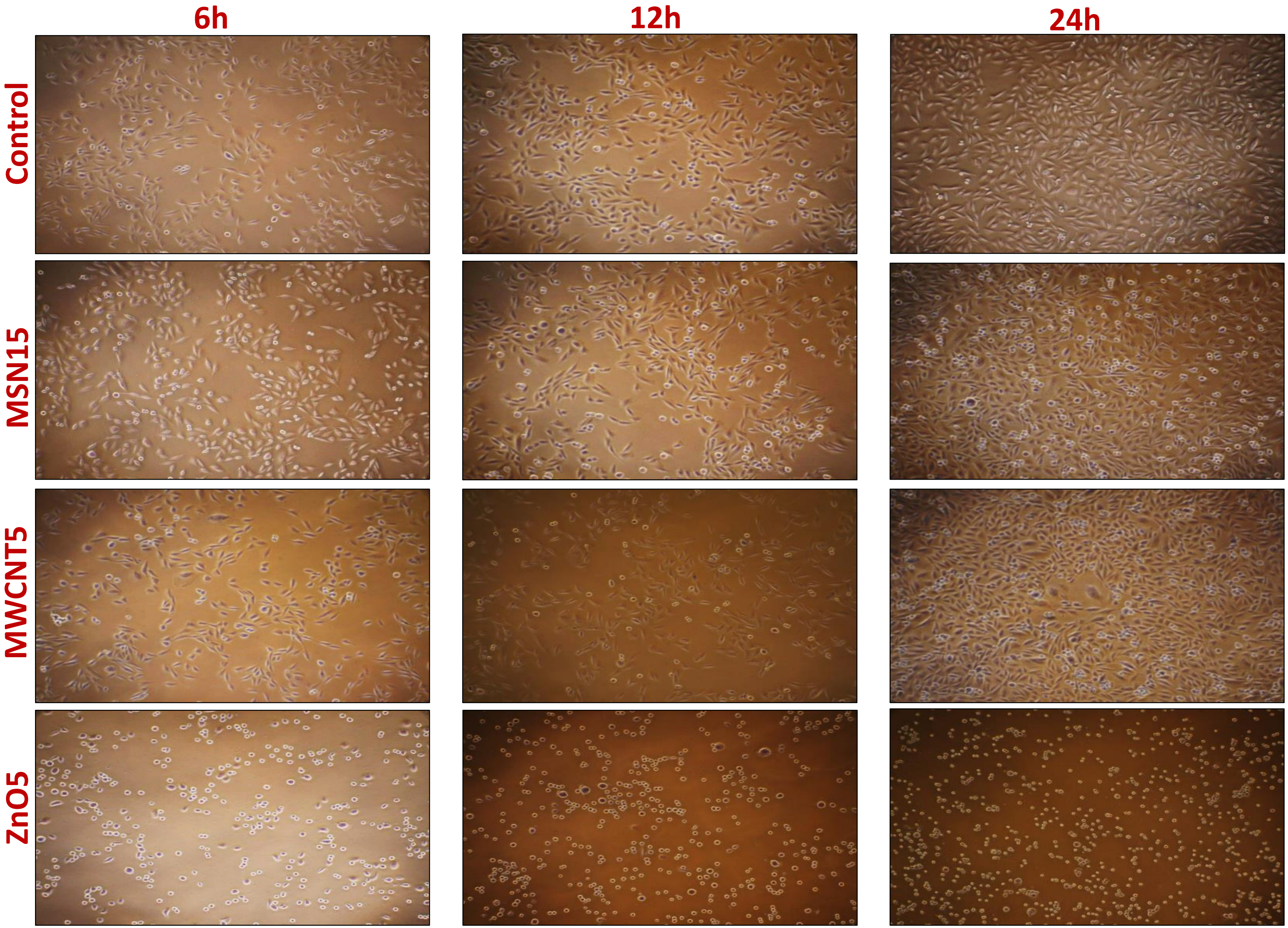

Figure S3: Morphological changes in CHO-K1 cells under bright field microscope. A. 100X magnification.

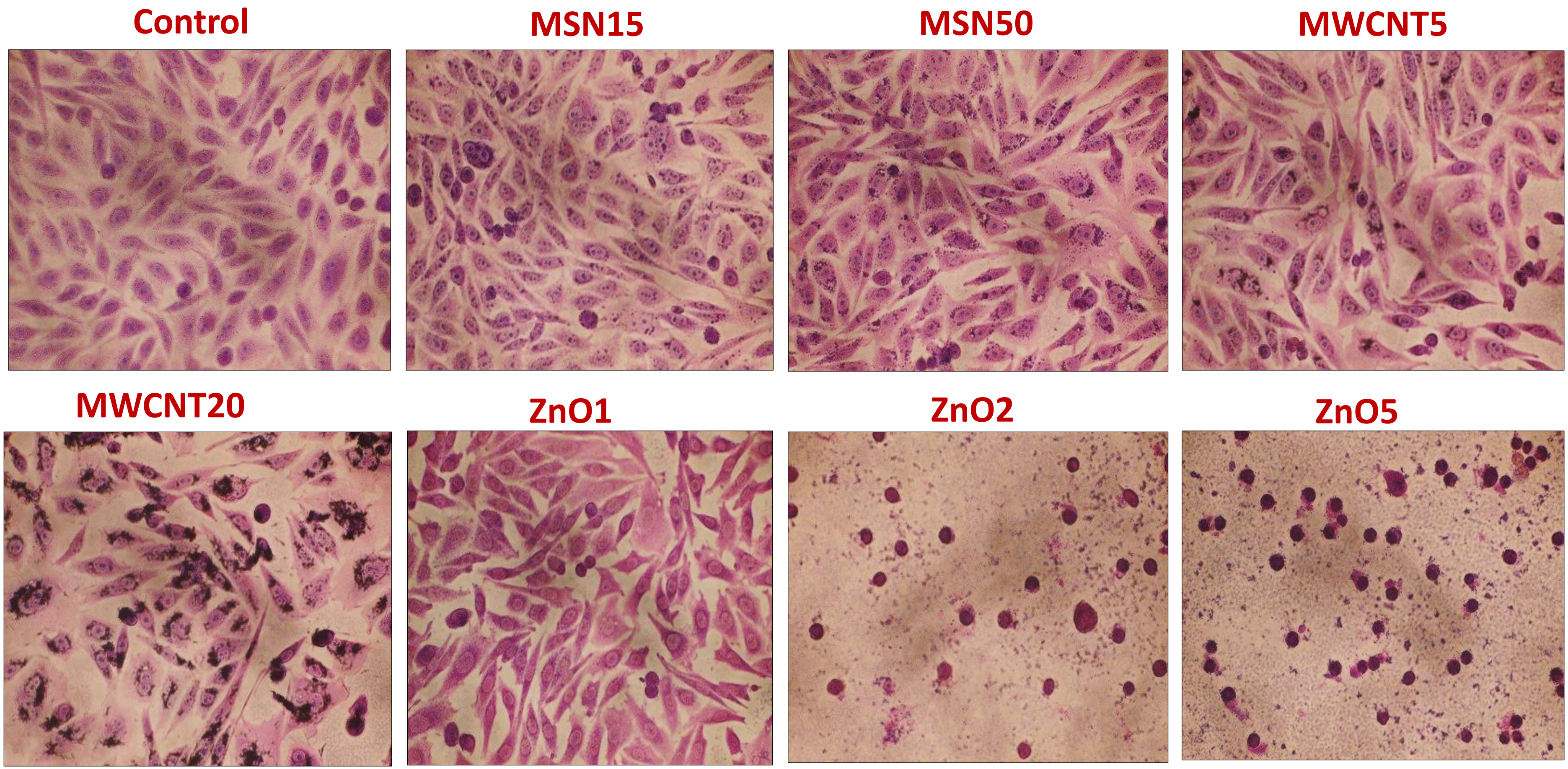

Figure S4: Morphological changes in CHO-K1 cells under bright field microscope. Effect of nanomaterials on cell morphology of CHO-K1 cells after MSN, MWCNT and ZnO NPs treatment. CHO-K1 cells were seeded in 6-well plates and nanomaterials for 24 hours for the stabilization of cells. CHO-K1 cells treated with MSN (15 and 50 µg/ml), MWCNT (5 and 20 µg/ml) and ZnO NPs (1, 2 and 5 µg/ml) and control cells for 24 h. Photographs were taken after 24 h, stain with Wright stain. 400X magnification.

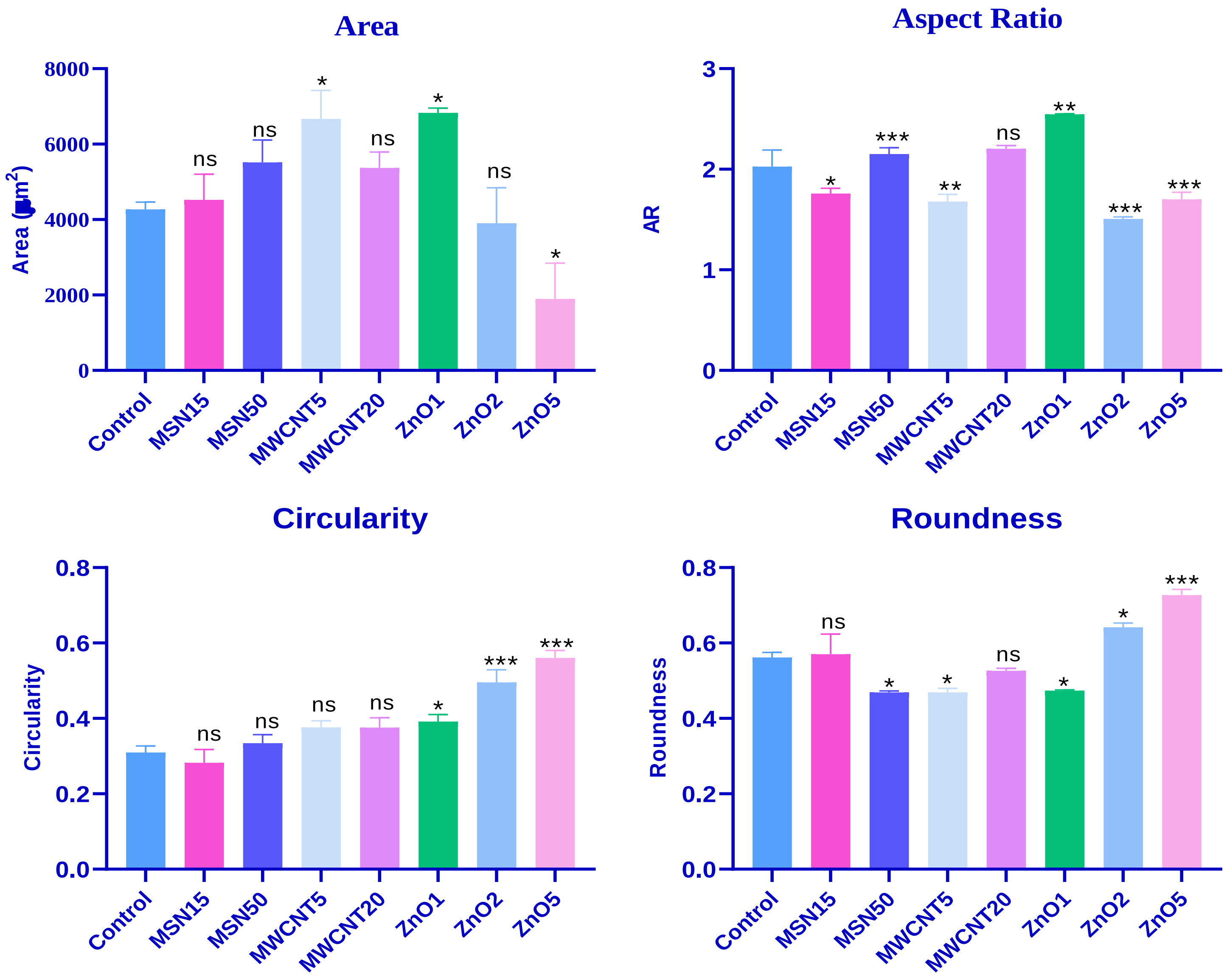

Figure S5. Morphological changes of CHO-K1 cells analyzed at 24 h post-treatment of MSN, MWCNT and ZnO NPs. **(A)** Area, **(B)** Aspect Ratio (AR), **(C)** Circularity and **(D)** Roundness measurements were taken.

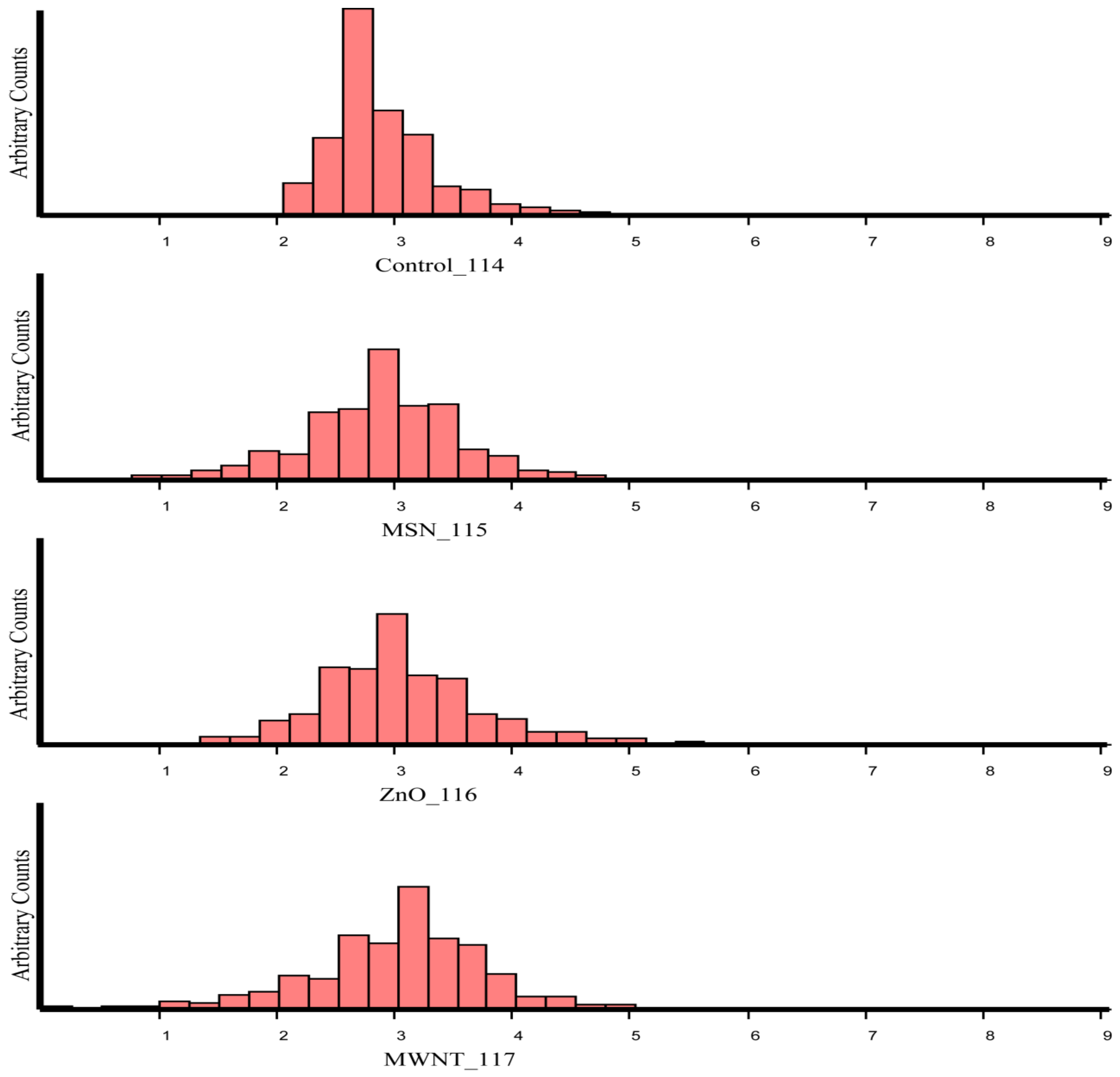

Figure S6: Histogram analysis: Total intensity count for the reporter ions identified per channel labelled. 114 label for the control, 115 is MSN treated, 116 is ZnO treated, and 117 is MWCNT treated. All the protein identified in the channel followed the normal distribution. Each bin the histogram represent total number of counts for log2 transformed intensities.

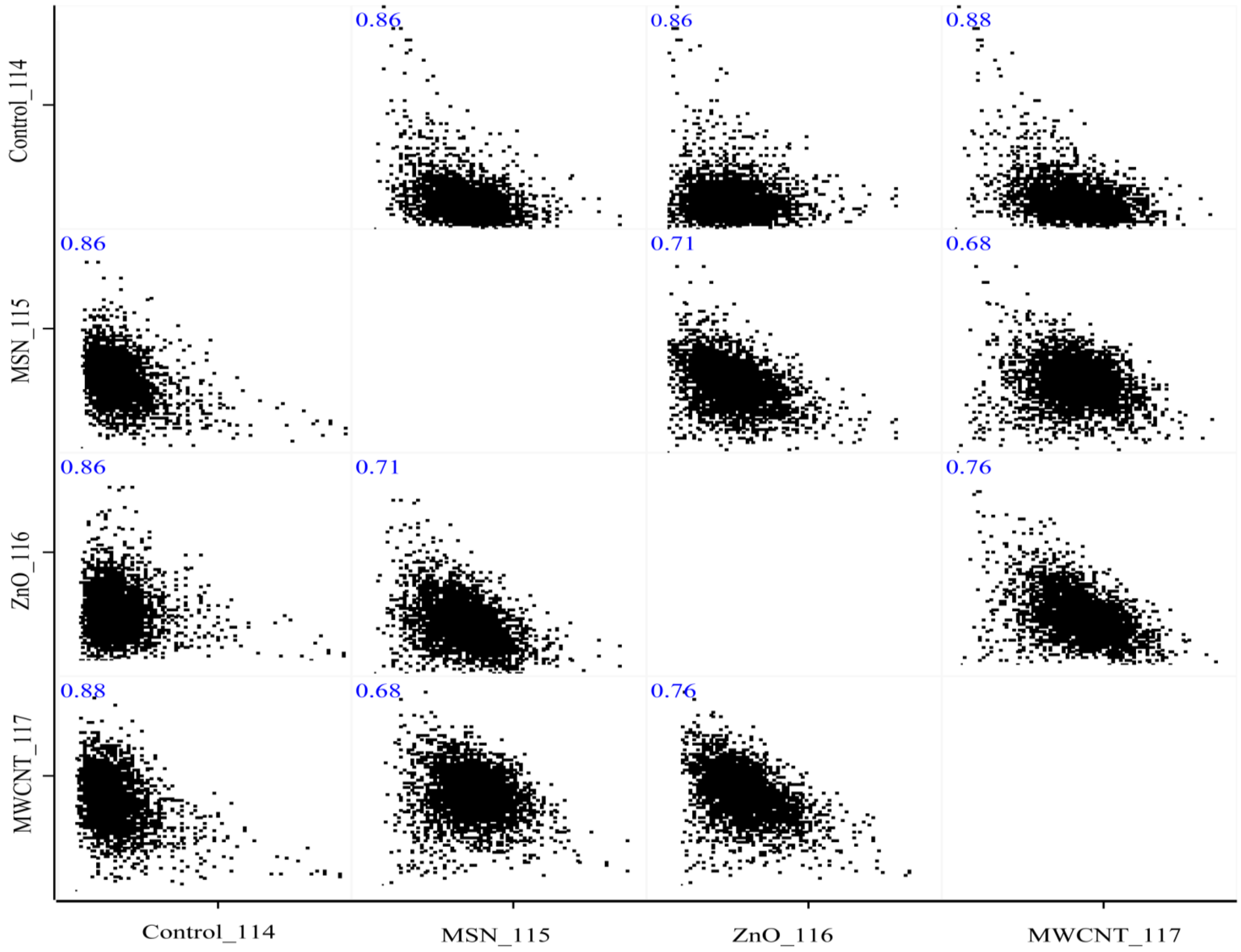

Figure S7: Multi Scatter plot analysis: All the identified reporter intensities were compared for the correlation among each other. The data represent high correlation among the nano-particle treatment. All the Pearson comparison values were represented in the left top corner of the scatter plot.

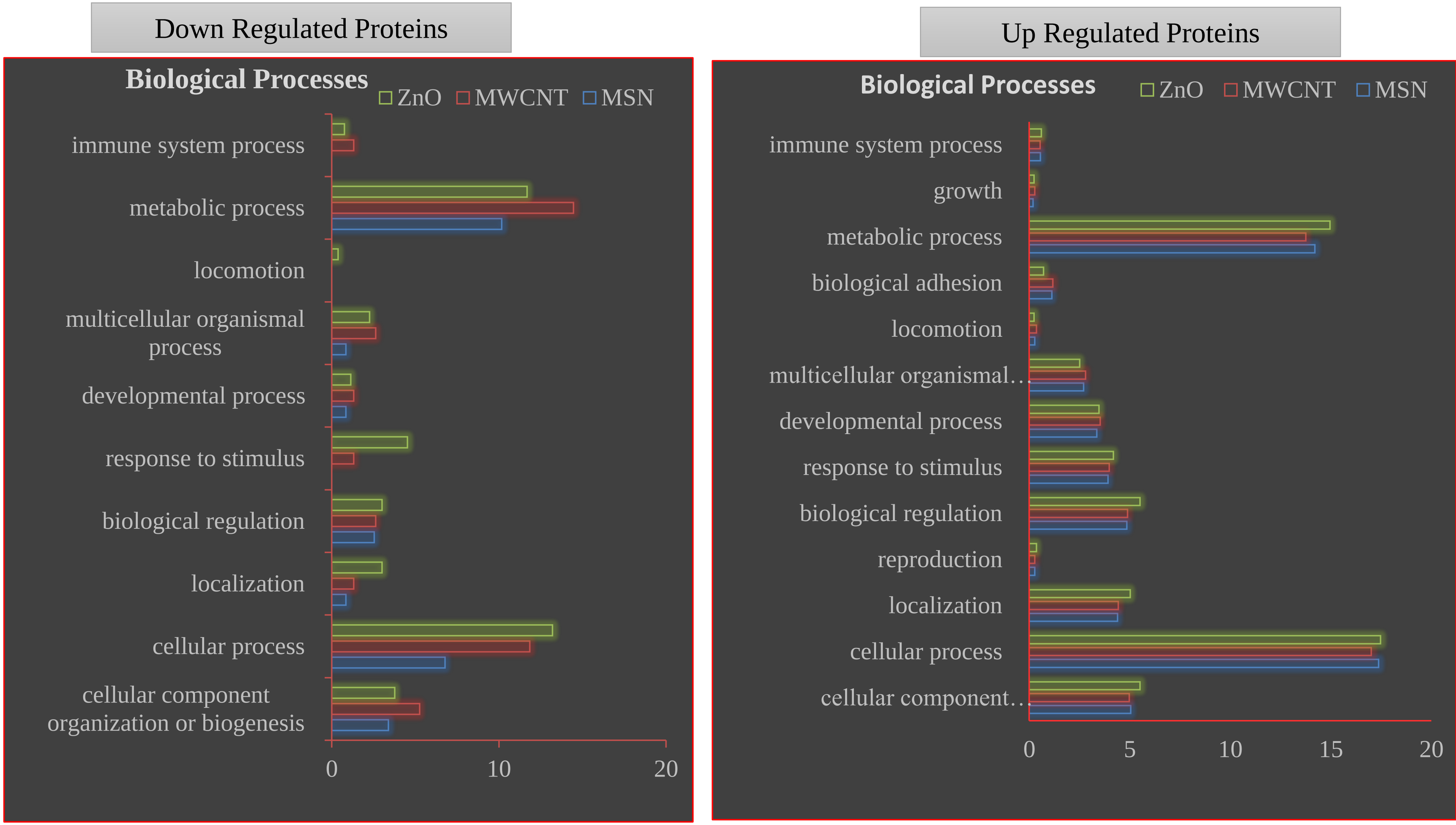

Figure S8: Gene Ontology analysis. Only differentially regulated proteins were subject to Gene Ontology (GO) analysis. These pictures show the up- and down-regulated GO terms for biological processes of the proteins. The blue bars represent proteins regulated by MSN NPs, red bars represent proteins regulated by MWCNT and the green bars represent proteins regulated by ZnO NPs.

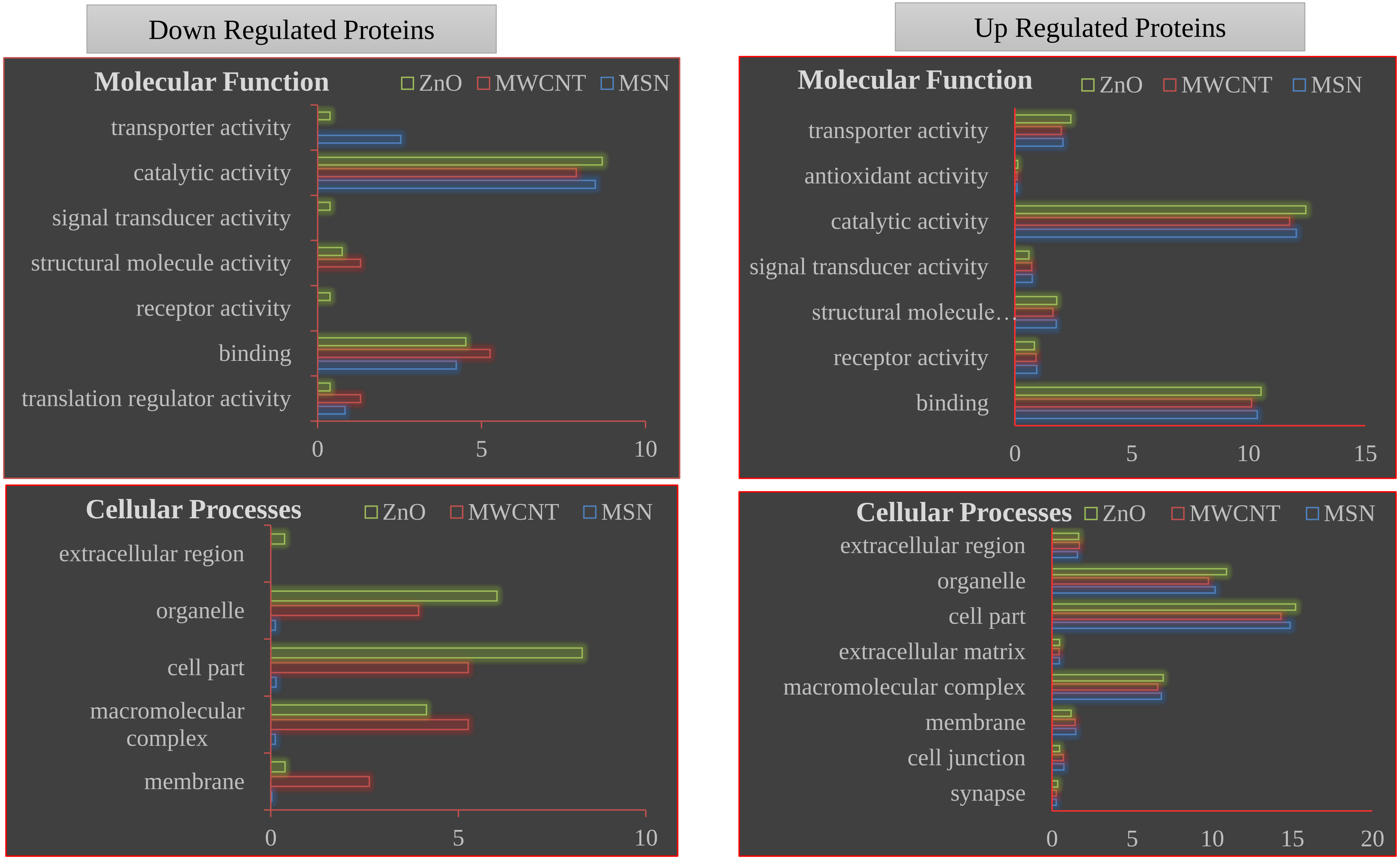

Figure S9: Gene Ontology analysis. Only differentially regulated proteins were subject to Gene Ontology (GO) analysis. These pictures show the up- and down-regulated GO terms for molecular function and cellular processes of the proteins. The blue bars represent proteins regulated by MSN NPs, red bars represent proteins regulated by MWCNT and the green bars represent proteins regulated by ZnO NPs.

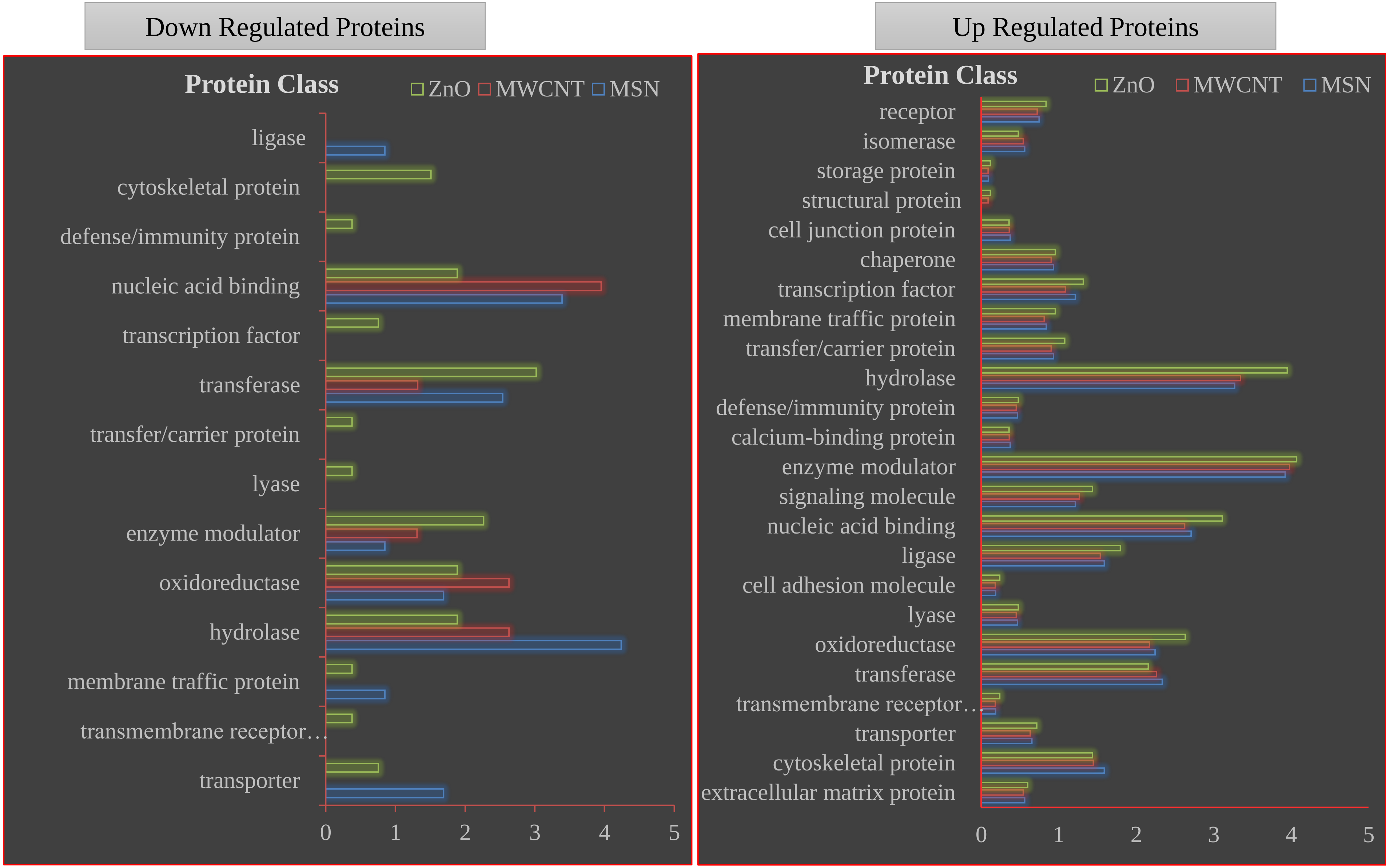

Figure S10: Gene Ontology analysis. Only differentially regulated proteins were subject to Gene Ontology (GO) analysis. These pictures show the up- and down-regulated GO terms for protein class of the proteins. The blue bars represent proteins regulated by MSN NPs, red bars represent proteins regulated by MWCNT and the green bars represent proteins regulated by ZnO NPs.

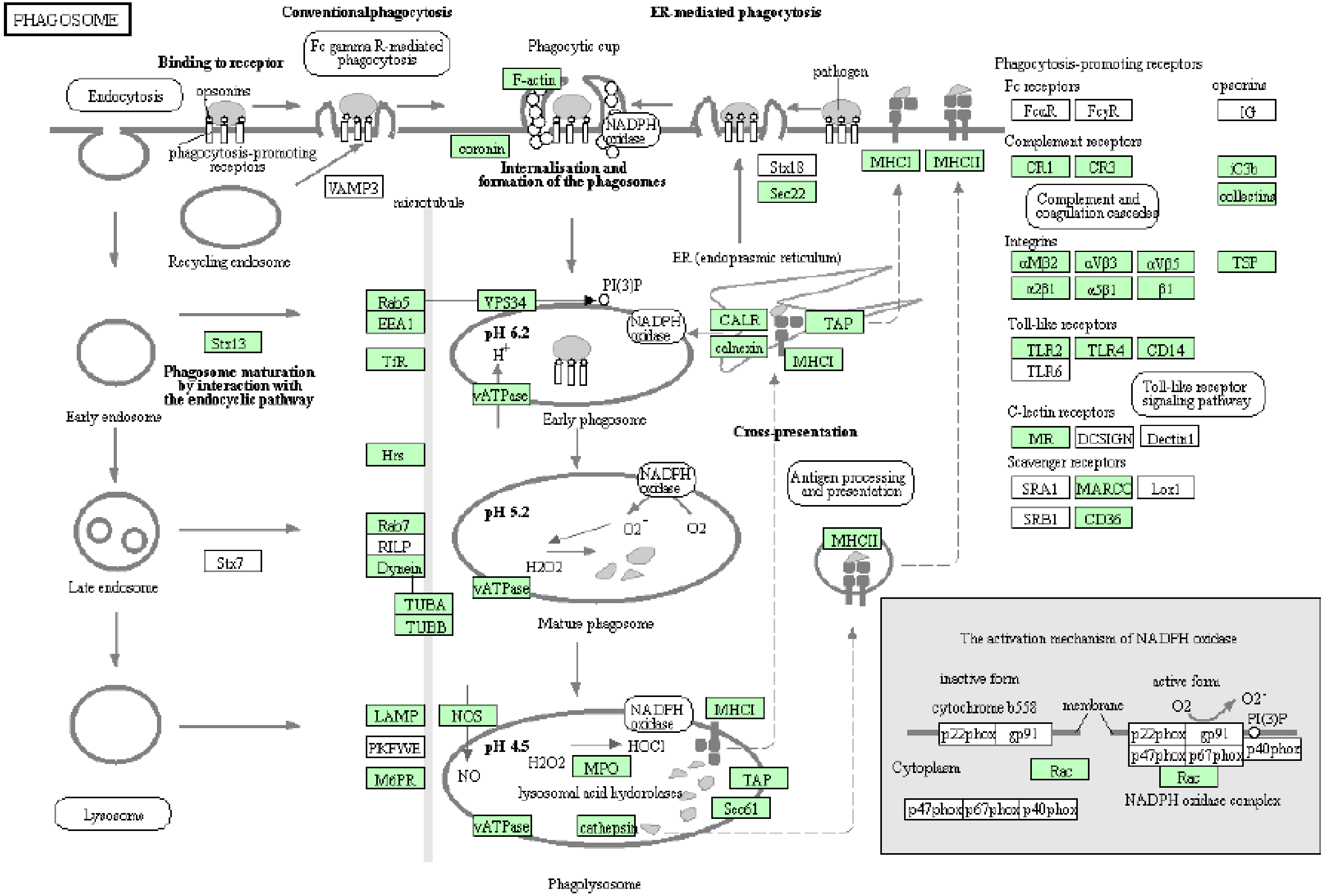

Figure S11: The phagosome KEGG pathway. Green rectangle means the identified proteins and white rectangle means reference pathway

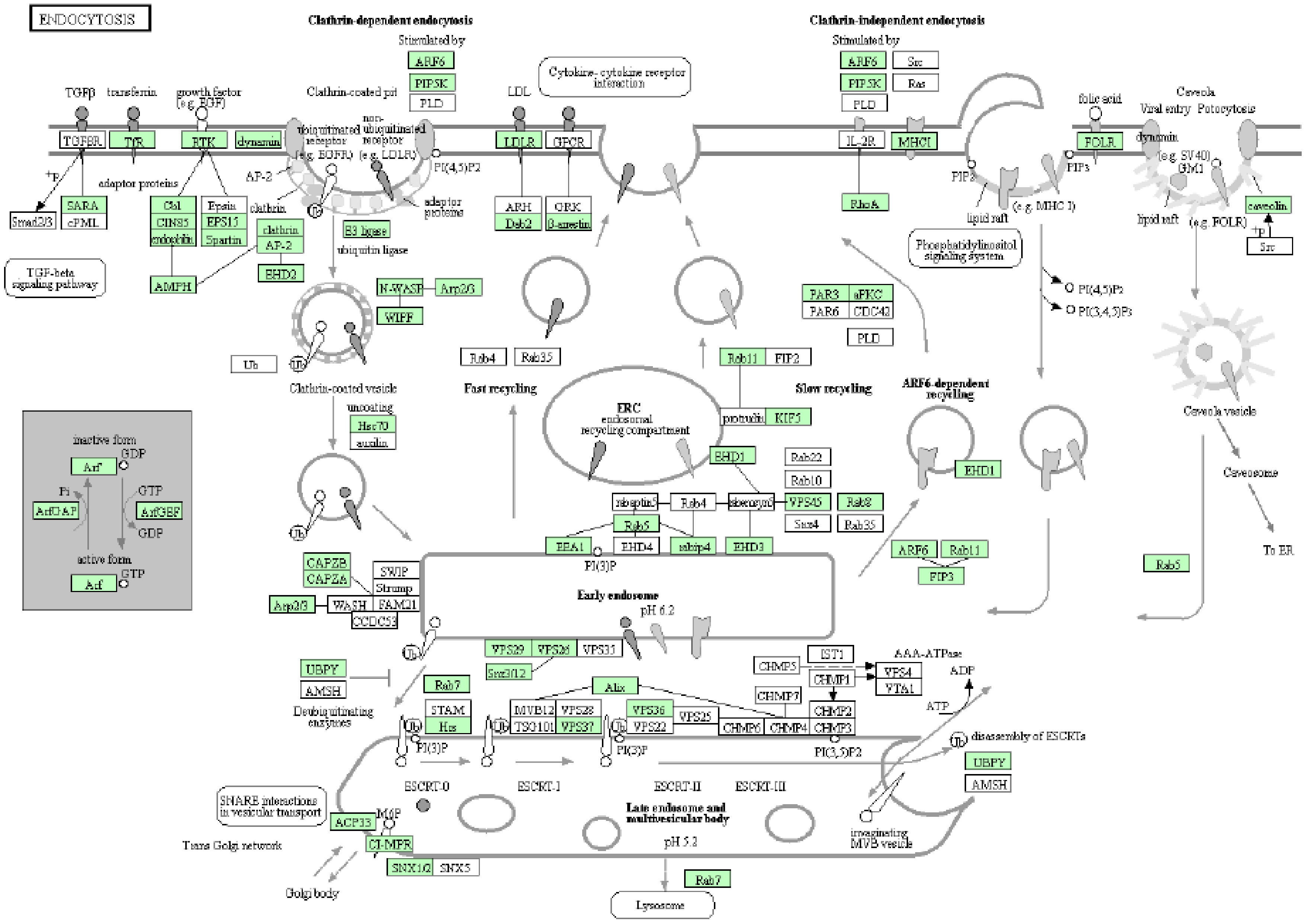

Figure S12: The endocytosis KEGG pathway. Green rectangle means the identified proteins and white rectangle means reference pathway

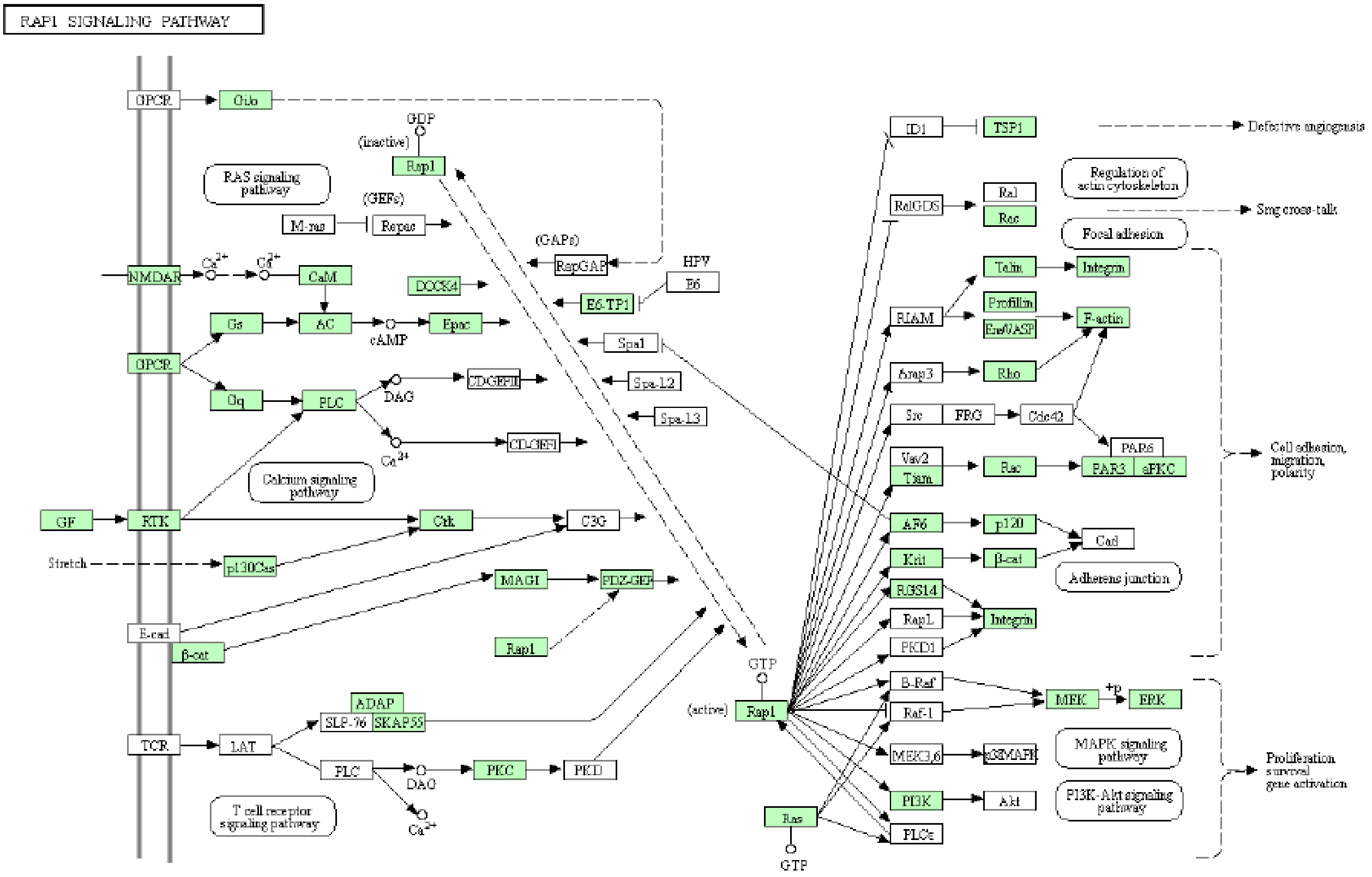

Figure S13: The Rap1 KEGG pathway. Green rectangle means the identified proteins and white rectangle means reference pathway

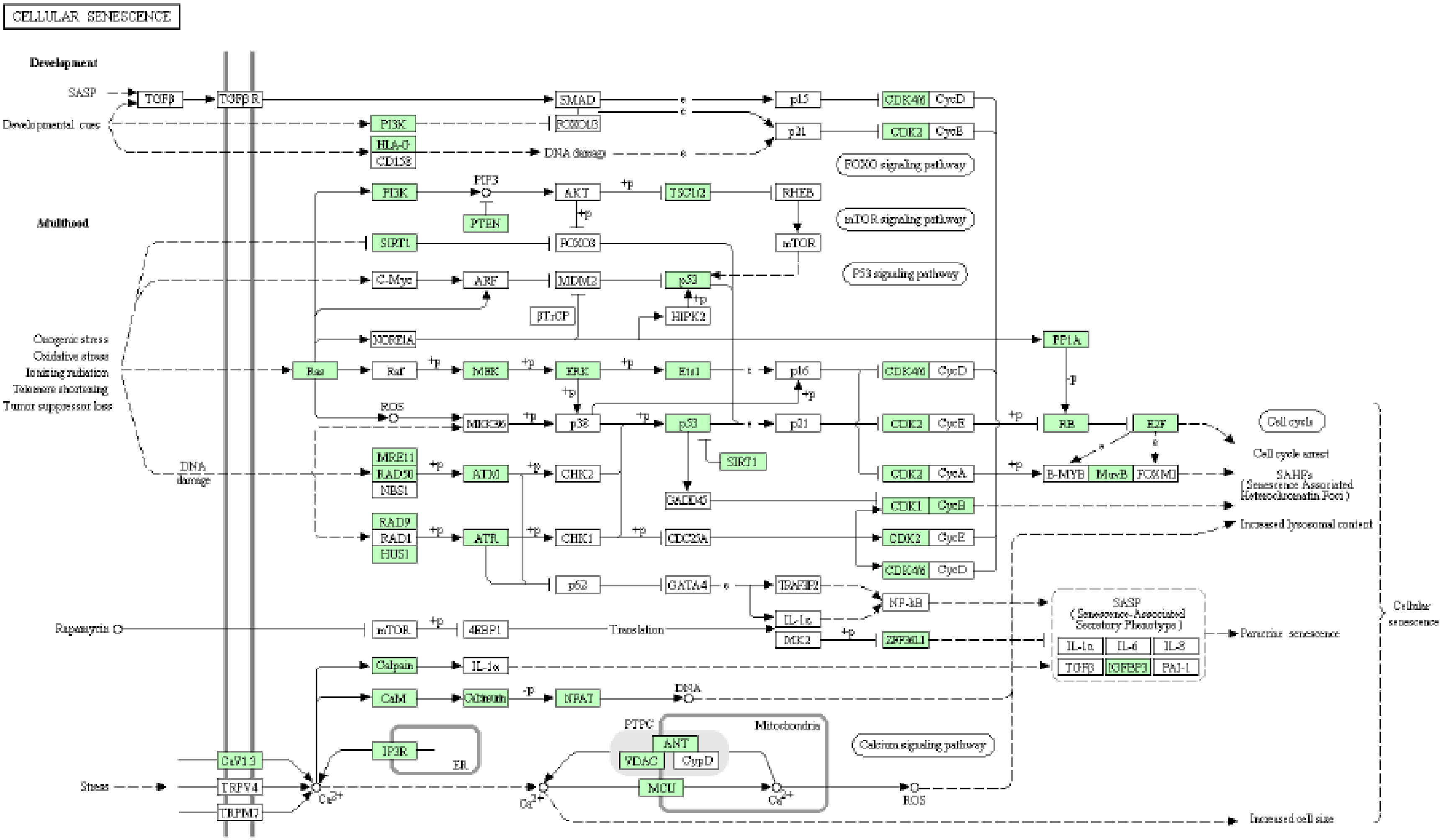

Figure S14: The cellular senescence KEGG pathway. Green rectangle means the identified proteins and white rectangle means reference pathway
