# Supplementary material for: MSN, MWCNT and ZnO nanoparticle-induced CHO-K1 cell polarisation is linked to cytoskeleton ablation
Source: J Nanobiotechnology. 2021 Feb 12;19:45. doi: 10.1186/s12951-021-00779-7 (PMC7881565; doi:10.1186/s12951-021-00779-7)
Supplement: Supplementary file 2 — Additional file 2: Table S1.1. Cytotoxicity of MSN, MWCNT and ZnO NPs in terms of mitochondrial activity by MTT and WST-8 assay. Table S1.2. Cytotoxicity of MSN, MWCNT and ZnO NPs in terms of membrane damage by LDH release assay. Table S1.3. Cytotoxicity of MSN, MWCNT and ZnO NPs in terms of viability by Trypan blue uptake assay. Table S1.4. Cytotoxicity of MSN, MWCNT and ZnO NPs in terms of morphology altered. [file 12951_2021_779_MOESM2_ESM.docx]

**Table S1.1: Cytotoxicity of MSN, MWCNT and ZnO NPs in terms of mitochondrial activity by MTT and WST-8 assay**

|  | **MTT Assay** | | | **WST-8 Assay** | | |
| --- | --- | --- | --- | --- | --- | --- |
| **Conc. μg/mL** | **MSN** | **MWCNT** | **ZnO NPs** | **MSN** | **MWCNT** | **ZnO NPs** |
| 0 | 100 | 100 | 100 | 100 | 100 | 100 |
| 1 | 91.65±7.262 | 93.58±4.889 | 81.74±3.261 | 95.53±0.2682 | 98.22±0.1602 | 87.43±0.0255 |
| 2 | 92.18±1.868 | 72.97±4.125 | 77.29±1.788 | 93.91±1.577 | 98.15±0.2309 | 76.71±0.9040 |
| 5 | 87.89±1.580 | 54.51±6.509 | 23.92±4.575 | 90.20±0.9443 | 95.43±0.6901 | 21.44±0.2298 |
| 10 | 79.79±4.187 | 42.65±8.997 | 16.35±0.5004 | 94.91±1.271 | 92.63±1.711 | 19.80±0.9885 |
| 20 | 79.00±0.6400 | 18.72±3.779 | 10.62±1.693 | 94.16±2.906 | 85.99±0.8318 | 13.37±0.2207 |
| 50 | 55.28±4.443 | 11.28±4.625 | 2.833±10.18 | 84.67±0.6338 | 70.12±0.9097 | 18.86±0.1891 |
| 100 | 47.05±5.551 | 12.46±3.118 | - | 82.99±0.8563 | 56.57±2.025 | 14.83±2.230 |

Data from 3 trials each were done in triplicate; Figures quoted as percent mean ± SD

**Table S1.2: Cytotoxicity of MSN, MWCNT and ZnO NPs in terms of membrane damage by LDH release assay**

| **Conc. μg/mL** | **MSN** | **MWCNT** | **ZnO NPs** |
| --- | --- | --- | --- |
| 0 | 0.0 | 0.0 | 0.0 |
| 0.5 | 0.7678±0.4534 | 2.243±1.466 | 2.213±0.4183 |
| 1 | 1.419±0.3191 | 4.174±1.914 | 4.142±1.868 |
| 2 | 3.688±1.916 | 4.950±2.618 | 8.415±0.1624 |
| 5 | 3.842±1.877 | 5.718±2.404 | 11.57±0.3817 |
| 10 | 5.094±1.443 | 10.14±0.2739 | 13.15±0.5158 |
| 20 | 6.704±1.902 | 22.24±1.416 | 30.48±3.277 |
| 50 | 7.982±1.573 | 35.00±2.185 | 80.60±1.080 |

Data from 3 trials each were done in triplicate; Figures quoted as percent mean ± SD

**Table S1.3: Cytotoxicity of MSN, MWCNT and ZnO NPs in terms of viability by Trypan blue uptake assay**

| **Conc. μg/mL** | **MSN** | **MWCNT** | **ZnO NPs** |
| --- | --- | --- | --- |
| 0 | 0.0 | 0.0 | 0.0 |
| 1 | 0.0 | 0.7800±0.0 | 1.326±0.0 |
| 2 | 7.274± 1.034 | 6.445± 1.589 | 9.282± 2.418 |
| 5 | 4.368± 0.0 | 16.56± 3.279 | 30.97± 2.363 |
| 10 | 1.612± 0.0 | 40.95± 1.404 | 83.07± 5.827 |
| 20 | 3.426± 1.905 | 104.5± 3.153 | 132.0± 10.09 |
| 50 | 12.86± 3.364 | 137.5± 8.171 | 225.8± 2.418 |

Data from 3 trials each were done in triplicate; Figures quoted as percent mean ± SD

**Table S1.4: Cytotoxicity of MSN, MWCNT and ZnO NPs in terms of morphology altered**

| **Parameter** | **Control** | **MSN15** | **MWCNT5** | **ZnO5 NPs** |
| --- | --- | --- | --- | --- |
| Area (µm^2^) | 4259±81.23 | 4149±465.8 | 6491±355.6 | 1664±451.7 |
| Aspect ratio | 2.009±0.0698 | 1.712±0.0518 | 1.695±0.0340 | 1.551±0.1013 |
| Circularity | 0.3140±0.0080 | 0.2594±0.0271 | 0.3806±0.0082 | 0.5363±0.0668 |
| Perimeter (µm) | 426.4±5.295 | 483.6±40.95 | 491.6±20.65 | 192.1±35.95 |
| Roundness | 0.5521±0.0112 | 0.6383±0.0176 | 0.6375±0.0131 | 0.7075±0.0290 |
| Solidity | 0.7726±0.0055 | 0.7708±0.0128 | 0.8118±0.0011 | 0.8321±0.03645 |

Data from 3 trials each were done in triplicate; Figures quoted as percent mean ± SD
